# Supplementary material for: Il4ra-independent vaginal eosinophil accumulation following helminth infection exacerbates epithelial ulcerative pathology of HSV-2 infection
Source: Cell Host Microbe. 2021 Apr 14;29(4):579–593.e5. doi: 10.1016/j.chom.2021.02.004 (PMC8062792; doi:10.1016/j.chom.2021.02.004)
Supplement: Document S1. Figures S1–S7 [file mmc1.pdf]

Cell Host & Microbe, Volume *29*

Supplemental information

Il4ra-independent vaginal eosinophil accumulation following helminth infection exacerbates epithelial ulcerative pathology of HSV-2 infection

Alisha Chetty, Matthew G. Darby, Pia M. Vornewald, Mara Martín-Alonso, Anna Filz, Manuel Ritter, Henry J. McSorley, Lindi Masson, Katherine Smith, Frank Brombacher, Matthew K. O'Shea, Adam F. Cunningham, Bernhard Ryffel, Menno J. Oudhoff, Benjamin G. Dewals, Laura E. Layland, and William G.C. Horsnell

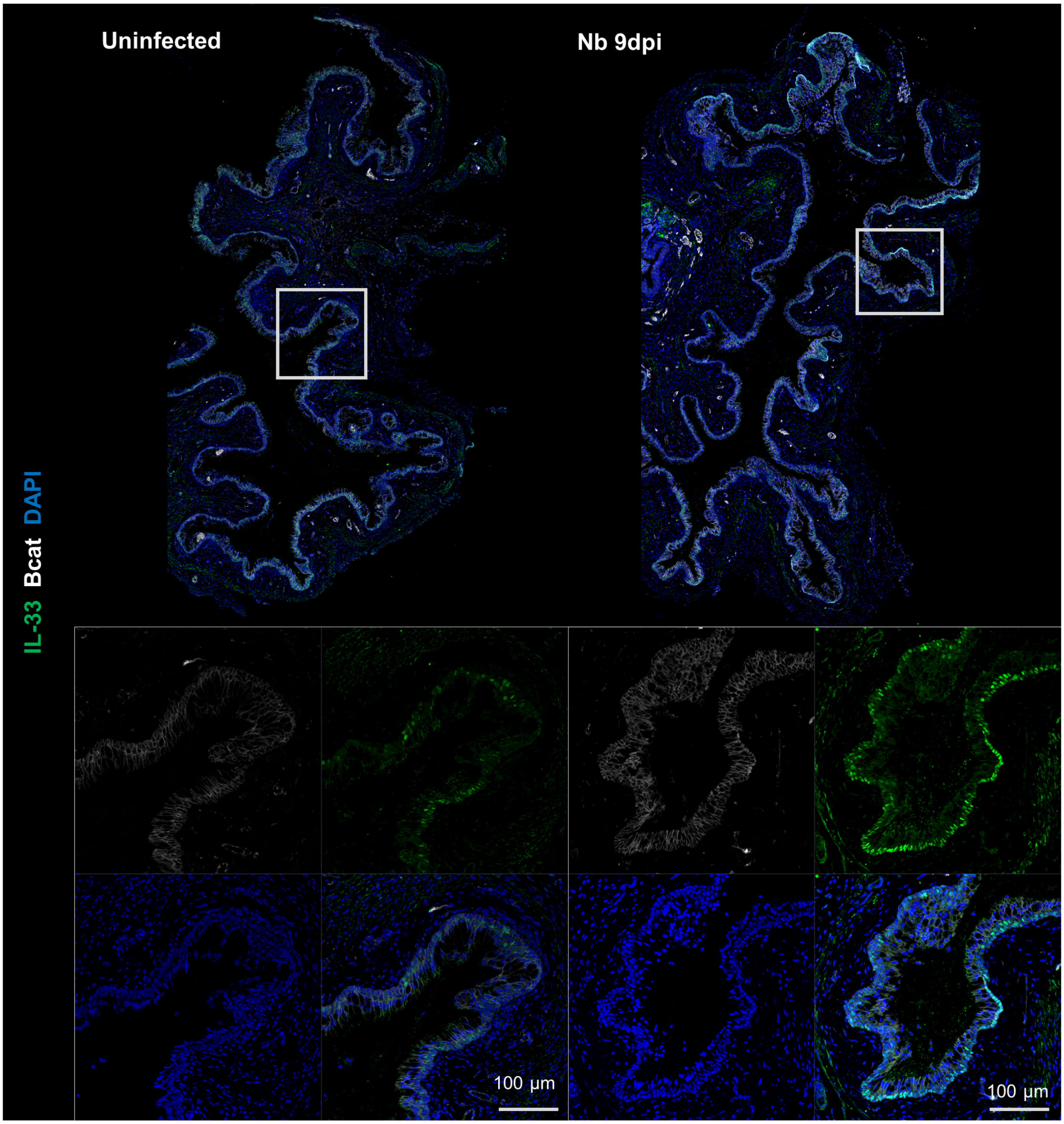

Figure S1. Vaginal epithelial cells are a major source of IL-33, Related to Figure 1: At day 9 post Nb infection (Nb 9dpi), vaginal tissue (n=2) was isolated for immunofluorescent (IF) staining for beta catenin (Bcat; white), IL-33 (green) and Hoechst 33342 (blue). White boxes indicate magnified sections. Data is representative of one experiment with 2 mice per group.

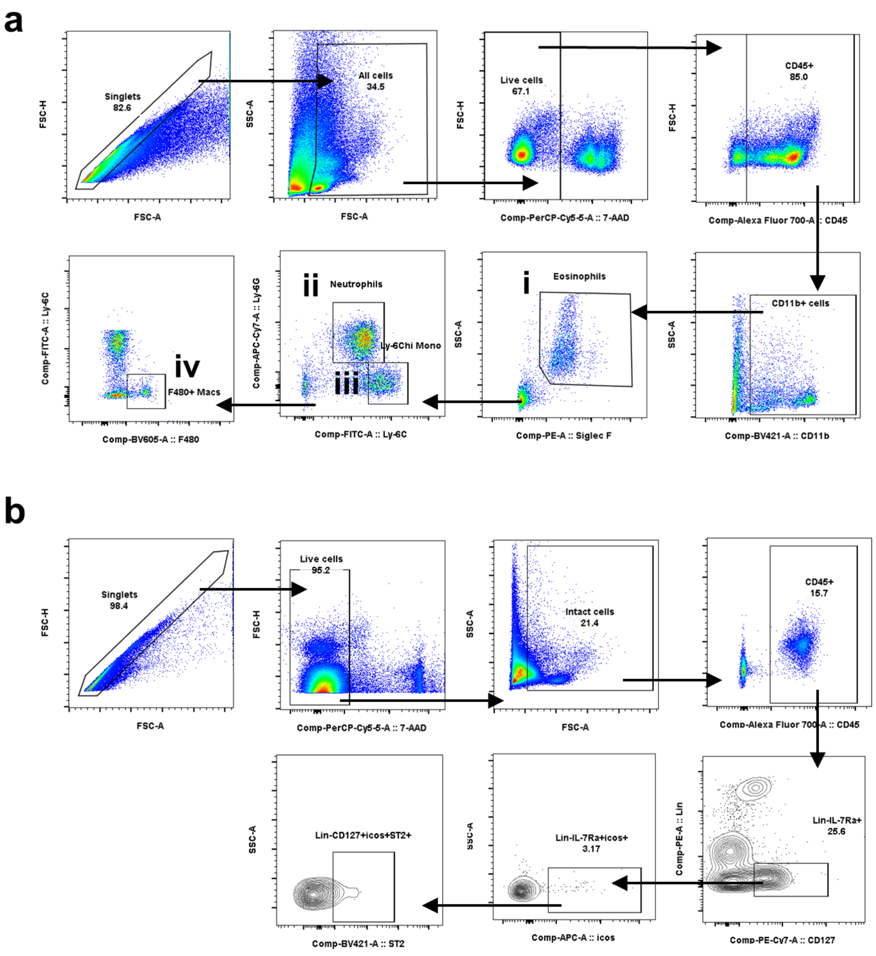

Figure S2. FGT and iLN flow cytometry gating strategy, Related to Star Methods: Flow cytometry: At day 3 post HSV-2 infection. FGT and iLNs were harvested and processed to a single cell suspension. Cells were stained with fluorochrome-conjugated antibodies and analysed by multi-colour flow cytometry. **(a)** The following gating strategy was used to identify **(i)** CD11b^{+}SigLec-F^{+}SSC^{hi} eosinophils, **(ii)** CD11b^{+}Ly-6G^{+} neutrophils, **(iii)** CD11b^{+}Ly-6C^{hi} monocytes and **(iv)** CD11b^{+}F480^{+} macrophages. **(b)** Gating strategy used to identify Lin^{-}IL-7Rα^{+}ICOS^{+}ST2^{+} ILC2s in the FGT and iLN.

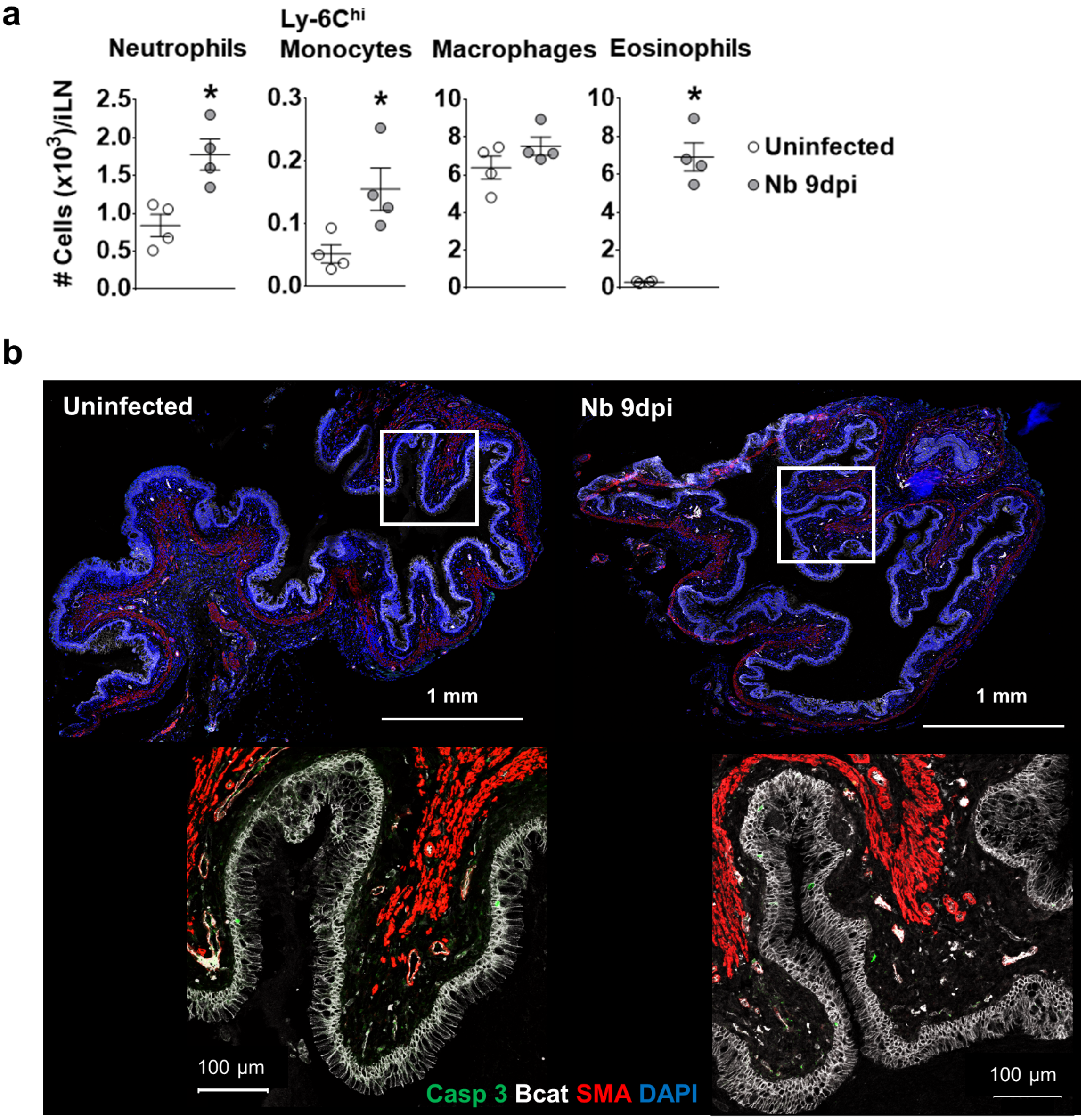

Figure S3. (a) Nb infection increases myeloid cell accumulation in the genital lymph nodes, Related to Figure 1: At 9dpi, myeloid cell populations in the iLN were analysed by flow cytometry to determine the numbers ($x10^3$) of neutrophils, Ly-6C^{hi} monocytes, macrophages and eosinophils in the iLN. Data is representative of two independent experiments with 4-5 mice per group (mean±sem). Statistical significance was calculated by Mann Whitney t test. *$p \leq 0.05$. **(b) Nb infection alone does not alter vaginal epithelial integrity, Related to Figure 1:** Female BALB/c mice were treated with 2 mg Depo Provera® (DP) 7 days prior to subcutaneous infection with Nb. At day 9 post Nb infection (Nb 9dpi), vaginal tissue (n=2) was isolated for immunofluorescent (IF) staining for beta catenin (Bcat), smooth muscle actin (SMA), 4',6-diamindino-2-phenylindole (DAPI) and cleaved caspase 3 (Casp 3). Images were taken using a 10x and 20x objective lens. White boxes indicate magnified sections. Data is representative of one experiment with 2 mice per group.

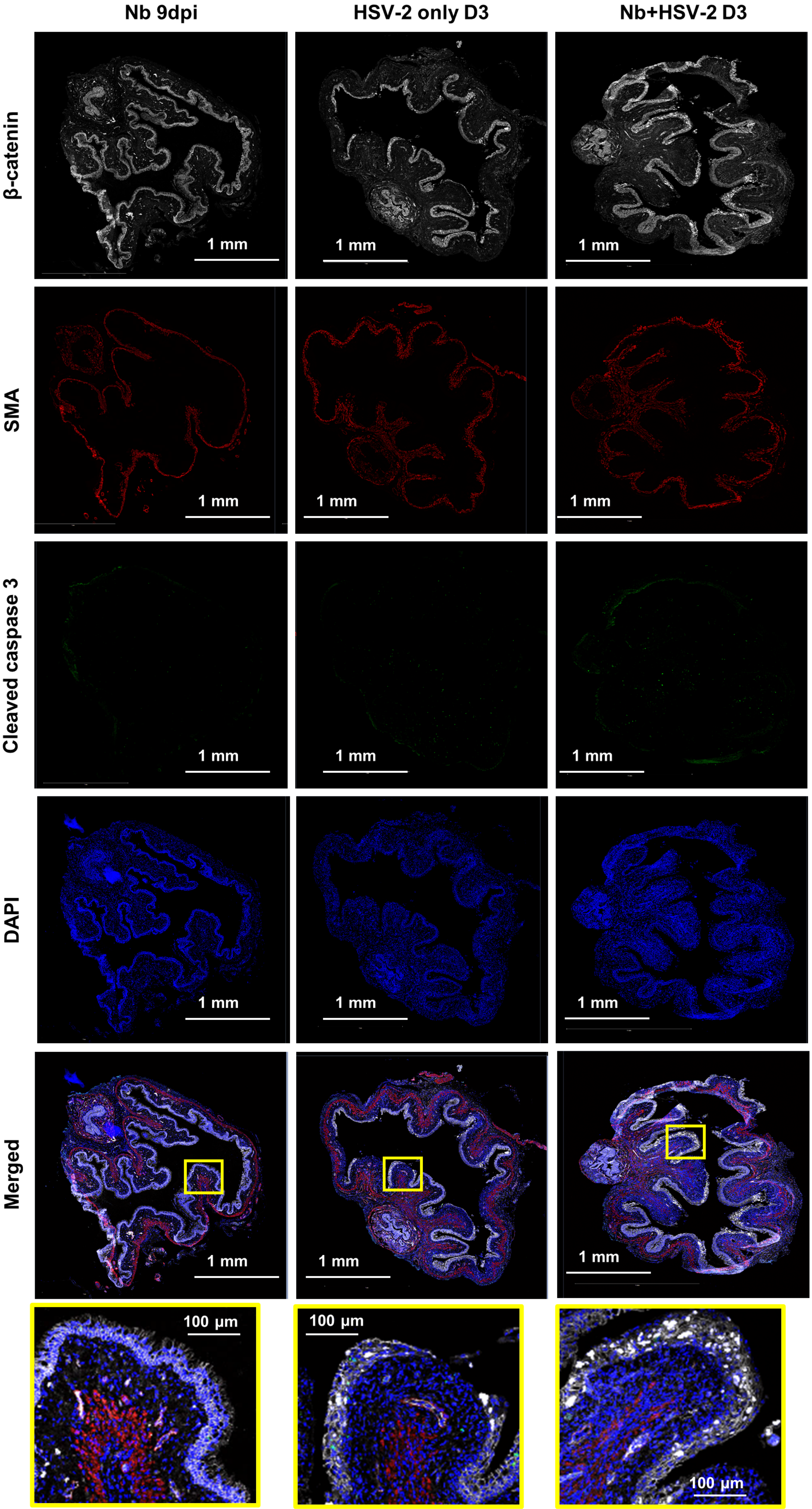

Figure S4. Virus-induced epithelial necrosis, rather than apoptosis, and early ulcer formation at day 3 post HSV-2 infection, Related to Figure 2: 7 days after Nb infection, mice were intravaginally infected with HSV-2. At day 3 post HSV-2 vaginal tissue were analysed by IF staining: beta catenin, smooth muscle actin (SMA), 4',6-diamindino-2-phenylindole (DAPI) and cleaved caspase 3. Images were taken using a 10x and 20x objective lens. Yellow boxes indicate magnified areas. Data is representative of one experiment with 4 mice per group.

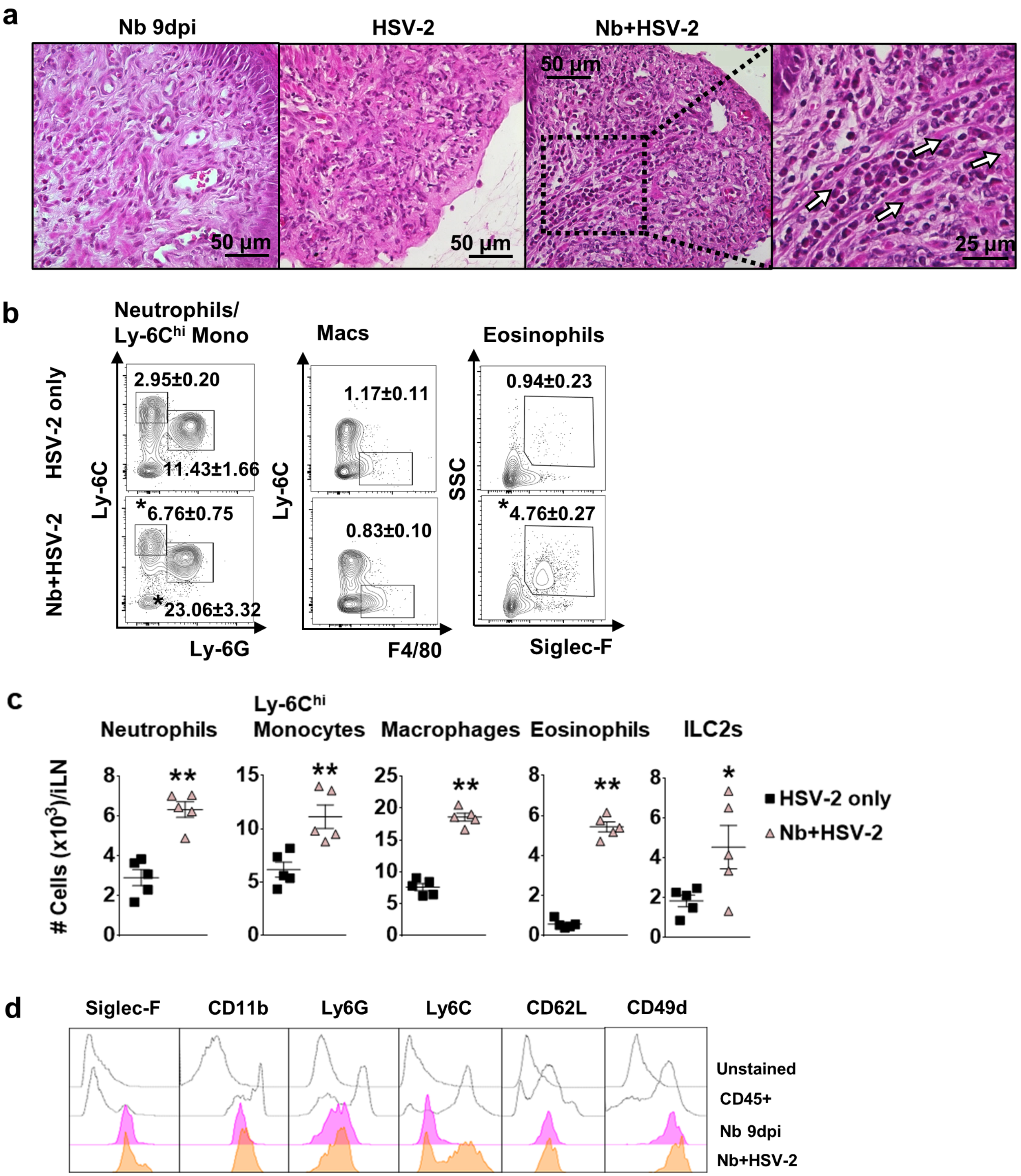

Figure S5. Prior Nb exposure increases myeloid cell inflammation in FGT and associated lymph nodes during subsequent HSV-2 infection, Related to Figure 3: At day 3 post HSV-2 vaginal tissue and iLNs were analysed by histology and flow cytometry, respectively. **(a)** Representative H&E-stained sections (n=4-5) of vaginal tissue of Nb only infected mice (day 9 PI), HSV-2 only and co-infected mice (day 3 post viral infection), displaying eosinophilic inflammation during co-infection (white arrows). **(b)** Frequencies (mean±sem) of neutrophils, Ly6C^{hi} monocytes, macrophages, and eosinophils in the FGT of HSV-2 only and Nb+HSV-2 mice at day 3 post virus infection. **(c)** Myeloid cell and ILC2 (Lin$^-$ IL-7Rα^+ICOS$^+$ST2$^+$) populations in the iLN of HSV-2 only and Nb+HSV-2 infected mice. **(d)** Representative histograms (n=4-5) of the mean florescence intensity (MFI) of Siglec-F, CD11b, Ly6G, Ly6C, CD62L and CD49d on FGT eosinophils in Nb 9dpi and Nb+HSV-2 mice. Data is representative of two independent experiments with 4-5 mice per group (mean±sem). Statistical significance was calculated by Mann Whitney t test. *$p \leq 0.05$, **$p \leq 0.01$.

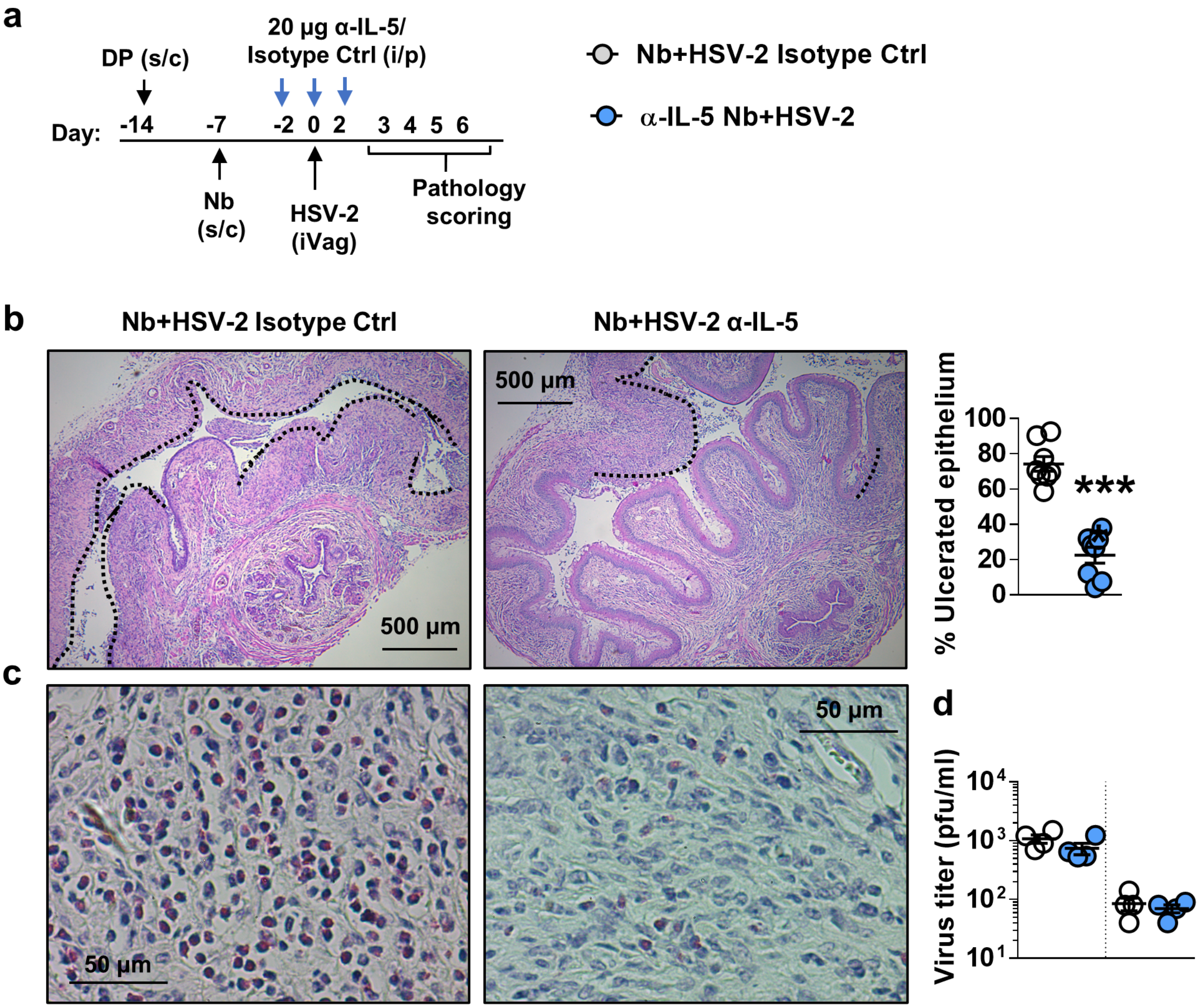

Figure S6. Anti-IL-5 antibody treatment protects against eosinophil-mediated Nb exacerbated pathology, Related to Figure 3: (a) Nb infected mice were treated with 20 µg α-IL-5 or isotype control, on day -2, 0 and 2 post HSV-2 infection. **(b)** Representative H&E-stained vaginal sections (n=4) of co-infected α-IL-5 or isotype treated mice at day 6 post HSV-2 infection. Images were taken at x50 magnification. Ulcerated vaginal epithelium is indicated by black dotted lines and qualified as percentage (%) of ulcerated epithelium. **(c)** Representative sections (n=4) of Sirius red-stained vaginal ulcers at day 6 post virus infection. Images were taken at x400 magnification. **(d)** Viral shedding was measured by plaque assay (PFU/ml) in day 3 and day 6 vaginal washes. Data is representative of two independent experiments with 4 mice per group (mean±sem). Statistical significance was calculated by Mann Whitney t test. *$p \le 0.05$, **$p \le 0.01$, ***$p \le 0.001$.

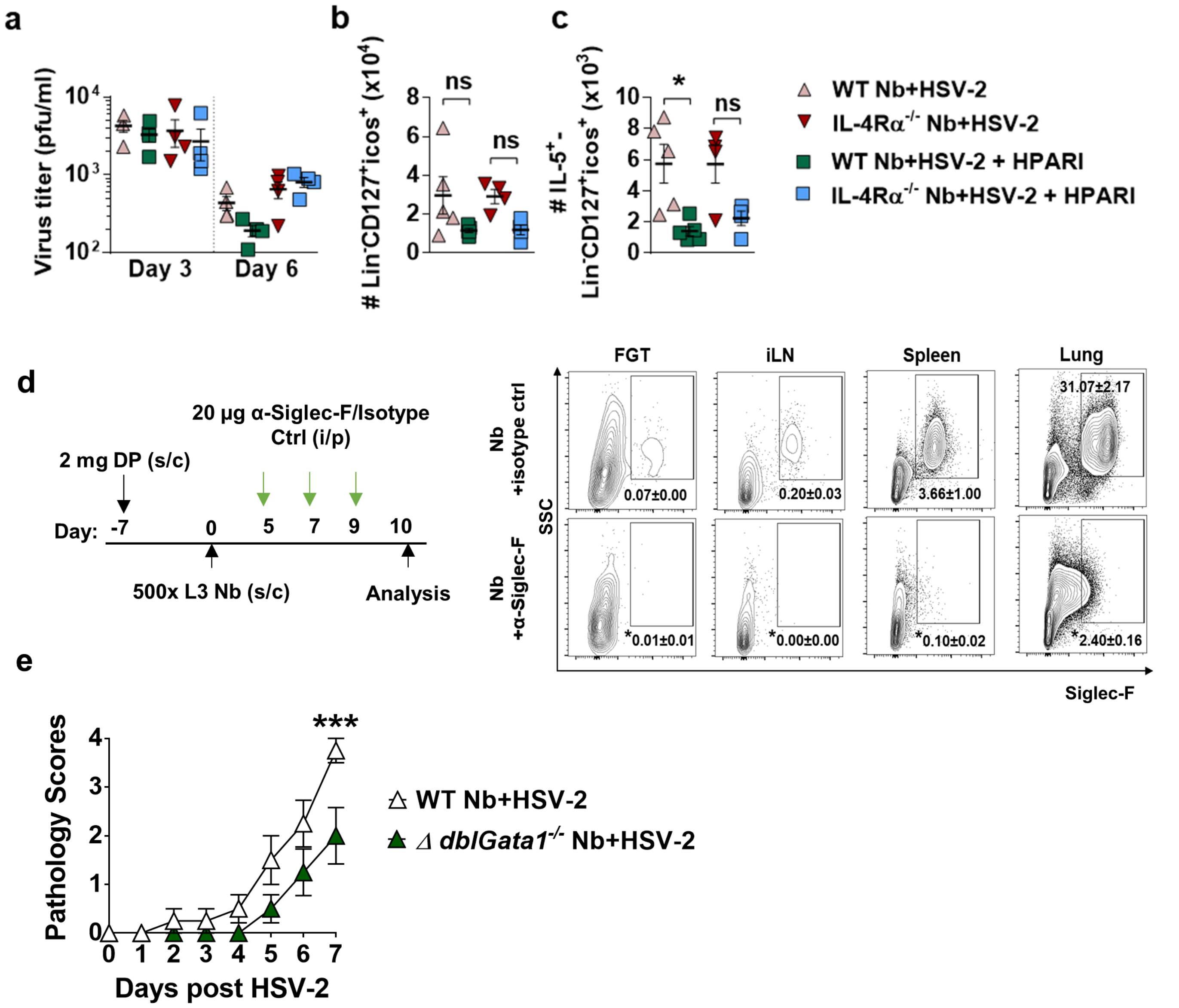

Figure S7. (a-c) IL-33 inhibition by vaginal HPARI treatment reduces IL-5 production by ILC2s, Related to Figure 5: (a) Viral shedding was measured by plaque assay (PFU/ml) in day 3 and day 6 vaginal washes. Data is representative of two independent experiments with 4 mice per group (mean±sem). Statistical significance was calculated by two-way ANOVA with Bonferroni correction for multiple comparisons. At day 6 post HSV-2 infection, FGT cells were analysed by flow cytometry: **(b)** numbers of Lin$^-$IL-7Rα$^+$ICOS$^+$ cells in the FGT and **(c)** the numbers of these cells, positive for intracellular IL-5. Data is representative of one experiment with 4-6 mice per group (mean±sem). Statistical significance was calculated by two-way ANOVA with Bonferroni correction for multiple comparisons. *$p \leq 0.05$. Related to Figure 5. **(d) Anti-Siglec-F antibody administration depletes tissue resident eosinophils, Related to Figure 6:** Nb infected mice were injected with 20 µg α-Siglec-F or isotype control, on day 5, 7 and 9 post infection. Representative flow plots (n=3) showing frequency (mean±sem) of CD11b$^+$SSC^{hi}Siglec-F$^+$ eosinophils in the FGT, iLN, spleen and lung, following antibody depletion. Data is representative of one experiment with 3 mice per group (mean±sem). Statistical significance was calculated by Mann Whitney t test. *$p \leq 0.05$. Related to Figure 6. **(e) Reduced HSV-2 pathology in co-infected *ΔdblGata1*$^{-/-}$ mice, Related to Figure 6:** Hormone synchronized WT and *ΔdblGata1*$^{-/-}$ co-infected mice were scored for virus-associated vaginal pathology following HSV-2 infection. Data is representative of one experiment with 3-4 mice per group (mean±sem). Statistical significance was calculated by two-way ANOVA with Bonferroni correction for multiple comparisons. ***$p \leq 0.001$.
