# Supplementary material for: Il4ra-independent vaginal eosinophil accumulation following helminth infection exacerbates epithelial ulcerative pathology of HSV-2 infection
Source: Cell Host Microbe. 2021 Apr 14;29(4):579–593.e5. doi: 10.1016/j.chom.2021.02.004 (PMC8062792; doi:10.1016/j.chom.2021.02.004)
Supplement: Document S2. Article plus supplemental information [file mmc2.pdf]

Article

Cell Host & Microbe

Il4ra-independent vaginal eosinophil accumulation following helminth infection exacerbates epithelial ulcerative pathology of HSV-2 infection

Graphical abstract

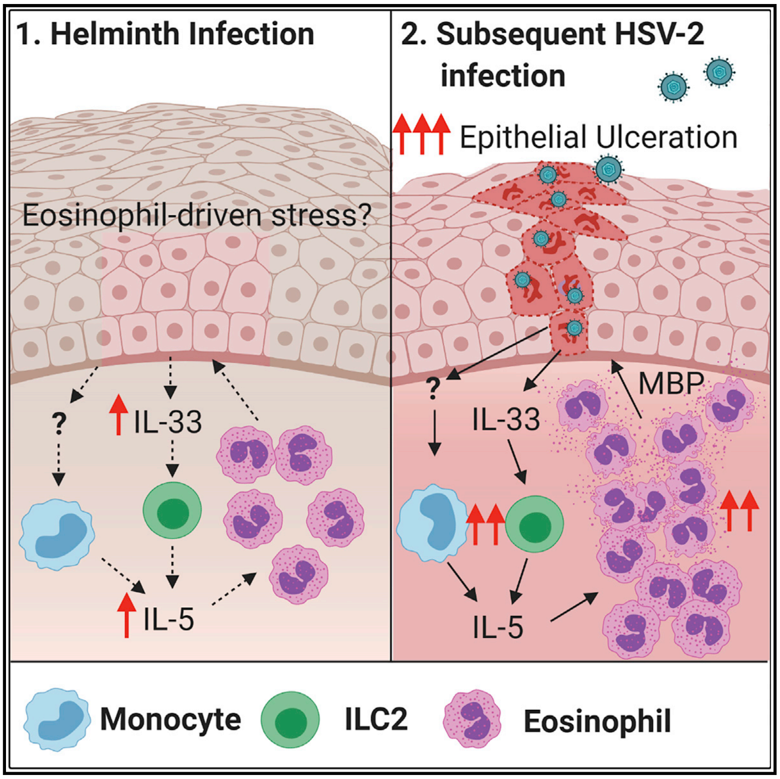

Highlights

- Helminth infection promotes pathology to vaginal HSV-2 infection
- Promoted pathology is IL-5, IL-33 mediated but *Il4rα* independent
- The enhanced vaginal epithelial ulceration is eosinophil driven

Authors

Alisha Chetty, Matthew G. Darby, Pia M. Vornewald, ..., Benjamin G. Dewals, Laura E. Layland, William G.C. Horsnell

Correspondence

laura.layland@microbiology-bonn.de (L.E.L.),
wghorsnell@gmail.com (W.G.C.H.)

In brief

Chetty et al. demonstrate that helminth infection systemically enhances vaginal pathology in subsequent HSV-2 infection. This is a result of an IL-5-promoted type 2 immunity expanding eosinophils in the vagina. These eosinophils cause an enhanced vaginal epithelial ulceration in co-infected mice.

Chetty et al., 2021, Cell Host & Microbe *29*, 579–593
April 14, 2021 Crown Copyright © 2021 Published by Elsevier Inc.
https://doi.org/10.1016/j.chom.2021.02.004

Cell Host & Microbe

CellPress
OPEN ACCESS

Article

Il4ra-independent vaginal eosinophil accumulation following helminth infection exacerbates epithelial ulcerative pathology of HSV-2 infection

Alisha Chetty,[1] Matthew G. Darby,[1] Pia M. Vornewald,[2] Mara Martín-Alonso,[2] Anna Filz,[3] Manuel Ritter,[3] Henry J. McSorley,[4] Lindi Masson,[5,6,7] Katherine Smith,[8] Frank Brombacher,[1,9] Matthew K. O'Shea,[10] Adam F. Cunningham,[10] Bernhard Ryffel,[11] Menno J. Oudhoff,[2] Benjamin G. Dewals,[12] Laura E. Layland,[3,13,*] and William G.C. Horsnell[1,11,14,15,*]

[1]Wellcome Centre for Infectious Diseases Research in Africa (CIDRI-Africa), Institute of Infectious Disease and Molecular Medicine (IDM), Department of Pathology, Division of Immunology, Faculty of Health Science, University of Cape Town, Cape Town 7925, South Africa
[2]CEMIR – Centre of Molecular Inflammation Research, Department of Clinical and Molecular Medicine, NTNU – Norwegian University of Science and Technology, 7491 Trondheim, Norway
[3]Institute for Medical Microbiology, Immunology and Parasitology (IMMIP), University Hospital Bonn (UKB), 53105 Bonn, Germany
[4]Division of Cell Signaling and Immunology, School of Life Sciences, University of Dundee, Wellcome Trust Building, Dow St, Dundee DD1 5EH, UK
[5]Institute of Infectious Disease and Molecular Medicine, University of Cape Town, Cape Town 7925, South Africa
[6]Centre for the AIDS Programme of Research in South Africa, Durban, South Africa
[7]Life Sciences Discipline, Burnet Institute, Department of Infectious Diseases, Monash University, Melbourne, VIC 3004, Australia
[8]Institute of Infection and Immunity, University of Cardiff, Cardiff CF14 3XN, UK
[9]International Centre for Genetic Engineering and Biotechnology, Cape Town 7925, South Africa
[10]Institute of Immunology and Immunotherapy, University of Birmingham, Birmingham, UK
[11]Laboratory of Experimental and Molecular Immunology and Neurogenetics (INEM), UMR 7355 CNRS-University of Orléans, 45000 Orléans, France
[12]Fundamental and Applied Research in Animals and Health (FARAH), Immunology-Vaccinology, Faculty of Veterinary Medicine (B43b), University of Liège, Liège, Belgium
[13]German Centre for Infection Research (DZIF), partner site, Bonn-Cologne, Bonn, Germany
[14]Institute of Microbiology and Infection, University of Birmingham, Birmingham, UK
[15]Lead contact
*Correspondence: laura.layland@microbiology-bonn.de (L.E.L.), wghorsnell@gmail.com (W.G.C.H.)
https://doi.org/10.1016/j.chom.2021.02.004

SUMMARY

How helminths influence the pathogenesis of sexually transmitted viral infections is not comprehensively understood. Here, we show that an acute helminth infection (*Nippostrongylus brasiliensis* [Nb]) induced a type 2 immune profile in the female genital tract (FGT). This leads to heightened epithelial ulceration and pathology in subsequent herpes simplex virus (HSV)-2 infection. This was IL-5-dependent but IL-4 receptor alpha (*Il4ra*) independent, associated with increased FGT eosinophils, raised vaginal IL-33, and enhanced epithelial necrosis. Vaginal eosinophil accumulation was promoted by IL-33 induction following targeted vaginal epithelium damage from a papain challenge. Inhibition of IL-33 protected against Nb-exacerbated HSV-2 pathology. Eosinophil depletion reduced IL-33 release and HSV-2 ulceration in Nb-infected mice. These findings demonstrate that Nb-initiated FGT eosinophil recruitment promotes an eosinophil, IL-33, and IL-5 inflammatory circuit that enhances vaginal epithelial necrosis and pathology following HSV-2 infection. These findings identify a mechanistic framework as to how helminth infections can exacerbate viral-induced vaginal pathology.

INTRODUCTION

Parasitic nematode infections and sexually transmitted viral infections (STVIs) occur at high rates in the same geographical locations, especially in low-middle income countries (LMICs) (Looker et al., 2015; WHO, 2002). Nematode infections do not normally colonize or transit the female genital tract (FGT), yet these infections have been associated with changes in both female fecundity (Blackwell et al., 2015) and FGT immunity (Chetty et al., 2020; Gravitt et al., 2016). For example, *Trichuris-trichiura*-infected women can display a distinct FGT type 2 cytokine profile associated with increased risk of human papillomavirus (HPV) infection (Gravitt et al., 2016). Nematode infections therefore appear to profoundly influence FGT biology and

Crown Copyright © 2021 Published by Elsevier Inc.
This is an open access article under the CC BY license (http://creativecommons.org/licenses/by/4.0/).

a critical consequence of this effect may be increased risk of infection and/or pathology from STVIs.

Nematode infections are known to alter host immunity in uncolonized tissue, these systemic effects can have important consequences for unrelated conditions at these sites. Effects can be complex and appear to be largely dependent on the context and biology of the helminth infection. For example, *Schistosoma-mansoni*- and *Nippostrongylus brasiliensis* (Nb)-induced IL-4 can promote CD8$^+$ T cell-driven control of murine gammaherpesvirus (Rolot et al., 2018), while *Heligmosomoides polygyrus* infection and *S. mansoni* egg challenge can impair host control of this virus (Reese et al., 2014). Natural and vaccine-mediated immunity to a pathogen can also be impaired; Nb infection reduces host control of non-typhoidal salmonella infection and induction of protective vaccination (Bobat et al., 2014). Additionally, canonical cellular responses to helminth infection, such as eosinophil recruitment, have been strongly associated with improved host control during *Mycobacterium tuberculosis* infection (O'Shea et al., 2018). Helminth infections therefore have diverse and critical influences on a host's ability to control unrelated infections. How helminths infections alter vaginal immunity and susceptibility to infections is currently not fully understood.

Genital herpes simplex virus (HSV)-2 is a common STVI worldwide, with high prevalence in helminth endemic regions and is associated with poorer male and female reproductive health (Freeman et al., 2006; Looker et al., 2017, 2015; Phipps et al., 2016). Initial host immunity to HSV-2 is classically driven by innate type 1 interferon (IFN)-promoted natural killer (NK) cell activity and subsequent IFN-γ, CD4$^+$, and CD8$^+$ T cell control (Lee et al., 2017; Milligan and Bernstein, 1997; Thapa et al., 2007). Type 1 responses are sensitive to downregulation by pre-existing or concurrent type 2/Th2 immune responses (Chang and Aune, 2007; Szabo et al., 1997; Wei et al., 2010). To date, direct demonstration of the consequences of helminth-induced type 2 immunity in the FGT, on immune control of HSV-2 infection, has not been described. Artificial induction of type 2 immunity in the FGT can increase pathogenesis of HSV-2 infection, for example, raised IL-33 levels were shown to enhance HSV-2 pathology and impair type 1 responses (Oh et al., 2016). Demonstrating whether nematode infections drive altered immunity to HSV-2 therefore remains an important and largely unanswered biological question that could have significant implications for female reproductive health. To gain insight into how helminth infections can alter STVI infections, this study uses a pre-clinical model to demonstrate that current and prior Nb infections initiate profound *Il4ra*-independent expansion of eosinophils in the FGT. This eosinophil expansion promotes increased vaginal epithelial ulceration following HSV-2 infection. These findings identify a nematode-induced systemic effect on immunity that promotes enhanced pathogenesis from a subsequent HSV-2 infection.

RESULTS

Nb infection results in a type 2 immune signature in the *FGT*

To test whether helminth infections can alter the underlying immune function of the FGT and associated lymph nodes, immune homeostasis in the FGT of hormone-synchronized mice was assessed at day 9 (immediately post-worm expulsion) and day 21 (12 days post-resolution) following infection (Figure 1A). In FGT of Nb-infected mice, raised levels of the epithelial alarmin IL-33 and canonical type 2 cytokines, IL-4 and IL-5, were found when compared with uninfected controls (Figure 1B). Raised IL-33 level is likely to be a consequence of epithelial stress, immunofluorescence (IF) staining of vaginal tissue for IL-33 and β-catenin (Bcat) confirmed vaginal epithelial cells to be the predominant source of IL-33 following Nb infection (Figure S1). Further histological analysis of FGT tissue demonstrated increased myeloid cell infiltration (Figure 1C) in Nb-infected mice when compared with uninfected mice. Sirius red staining indicated a significant element of this infiltrate were eosinophils (Figure 1D). Flow cytometric analysis of FGT myeloid cell populations according to the applied gating strategy (Figure S2) confirmed this observation. Nb-infected mice showed a significant increase in the proportions and numbers of eosinophils (CD11b$^+$SiglecF$^+$SSC^{hi}), a modest induction of Ly6C^{hi} inflammatory monocytes (CD11b$^+$Ly6C^{hi}Ly6G$_{lo}$), and a trend for increased neutrophils (CD11b$^+$Ly6G$^+$) in the FGT, in comparison with uninfected mice (Figure 1E). Comparative analysis of myeloid cells in the associated iliac lymph nodes (iLNs) also revealed increased numbers of eosinophils, Ly6C^{hi} monocytes, and neutrophils in mice 9 days post-infection (dpi) compared with uninfected controls (Figure S3A). The expanded FGT eosinophil population was maintained at 21 dpi (Figure 1E), indicating a long-term effect of Nb on FGT immunity.

Little data exist on eosinophil phenotypes in the FGT. To address this, we compared the expression of established markers of eosinophil function in the lung and FGT (Figure 1F). As expected, in the lung, we identified Siglec-F^{hi} and Siglec-F^{int} eosinophil populations that are representative of recruited and resident eosinophils (Mesnil et al., 2016). Relative expression of CD11b, CD62L, and CD49d on eosinophils in the lung and FGT was used to identify molecular characteristics of these populations (Grayson et al., 1998; Percopo et al., 2017; Veen et al., 1998; Walker et al., 1993). We found Nb-induced FGT eosinophils to be Siglec-F^{int}CD11b^{int}CD62L^{int}CD49d^{hi}, a phenotype distinct from Siglec-F^{hi} and ^{int} populations found at the site of infection (lung) (Figure 1F). Nb-induced FGT eosinophils expressed raised levels of the integrins CD11b and L-selectin (CD62L) relative to Siglec-Fint lung eosinophils combined with elevated expression of CD49d (Borchers et al., 2001; Grayson et al., 1998; Henderson et al., 1997; Nakajima et al., 1994). Together, this suggests that FGT eosinophils are predominantly a recruited population following Nb infection.

To identify changes in FGT epithelial integrity following Nb infection, IF staining for Bcat and cleaved caspase-3 (c-Casp-3) was carried out. This revealed equivalent epithelial integrity in vaginal tissue of uninfected and Nb 9 dpi mice (Figure S3B). Together these findings show that Nb infection induces expansion of myeloid cell populations in the FGT, and although this did not result in distinct histological changes to epithelium, raised levels of IL-33 in vaginal lavages and in the epithelium (Figure S1) suggests the presence of epithelial stress.

HSV-2 FGT pathology is exacerbated with prior Nb infection

To test whether Nb-associated immune changes in the FGT had a consequence on an unrelated infection, we infected mice

CellPress
OPEN ACCESS

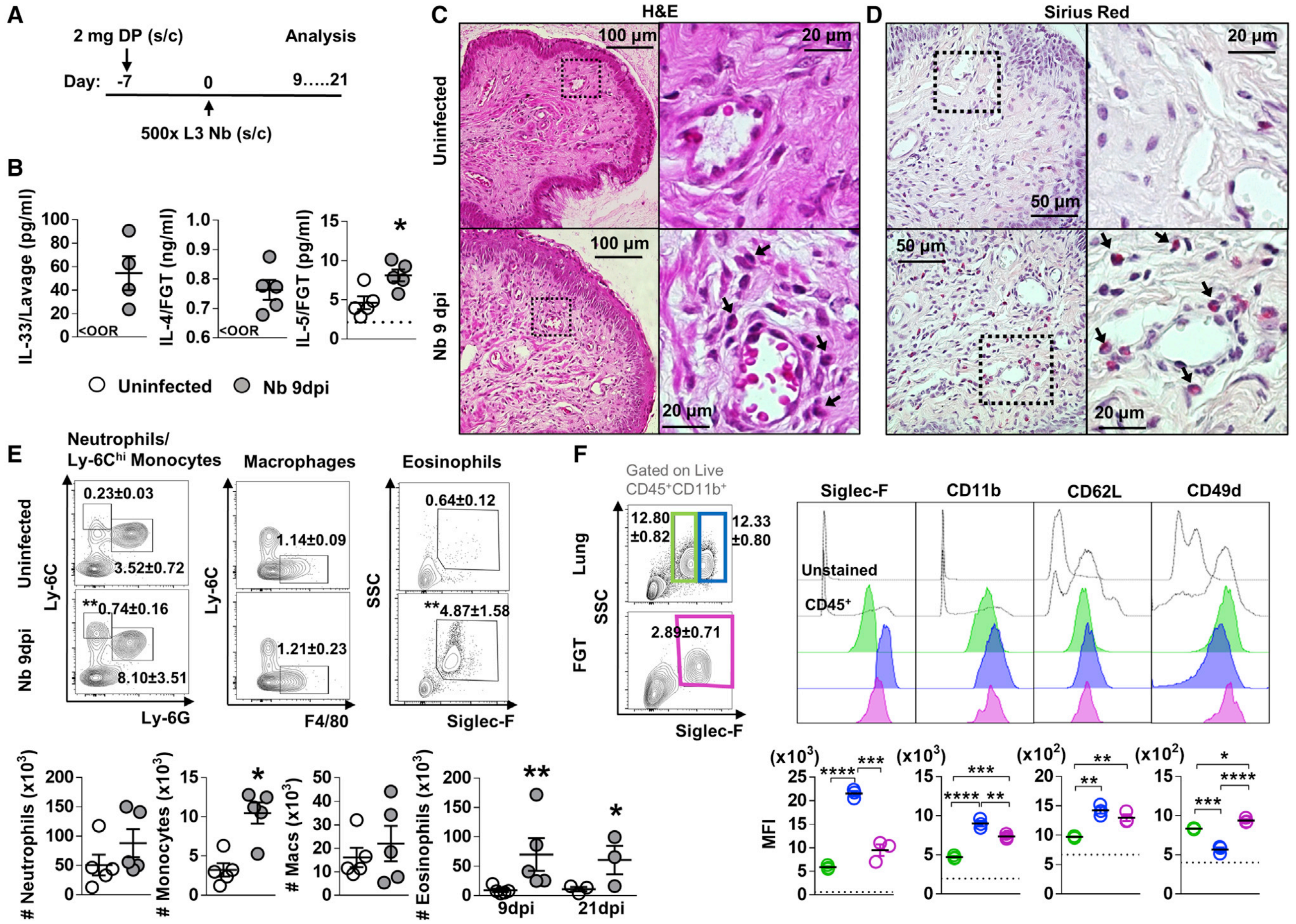

Figure 1. Influence of *N. brasiliensis* exposure on uncolonized FGT, increase in FGT eosinophils following Nb exposure

(A) Female mice were hormone-synchronized 7 days prior to Nb infection.

(B) At day 9 post-Nb infection (Nb 9dpi), levels of IL-33, IL-4, and IL-5 in FGT homogenates or lavages were assessed by ELISA or Luminex. <OOR: below detection range for ELISA. Dotted line represents lower limit of quantification (LLOQ) for Luminex analysis. Vaginal tissue was analyzed by (C and D) (C) hematoxylin and eosin (H&E) and (D) Sirius red staining. Representative images (n = 5) were taken at ×200, ×400, and ×1,000 magnification. Black arrows identify eosinophils.

(E) Frequencies (mean ± SEM) and numbers (×10^3) of neutrophils (CD11b$^+$Ly-6G$^+$), Ly-6C^{hi} monocytes (CD11b$^+$Ly-6C^{hi}), macrophages (CD11b$^+$F480$^+$), and eosinophils (CD11b$^+$Siglec-F$^+$SSC^{hi}) in the FGT of naive and Nb-infected mice.

(F) Mean fluorescence intensity (MFI) of Siglec-F, CD11b, Gr-1, CD62L, and CD49d on lung (green, blue) and FGT (pink) eosinophils at Nb 9 dpi. Dotted line represents the MFI of CD45$^+$ FGT cells. Data are representative of two independent experiments with 4–5 mice per group (mean ± SEM). Statistical significance was calculated by Mann-Whitney t test. *$p \leq 0.05$, **$p \leq 0.01$, ***$p \leq 0.001$, ****$p \leq 0.0001$.

intravaginally with HSV-2, 7 days after Nb infection (Figure 2A). Nb + HSV-2 co-infection resulted in a step shift elevation in genital pathology, with raised vaginal inflammation from day 3 post-HSV-2 infection and increased genital ulceration by day 6, when compared with HSV-2-only-infected mice (Figure 2B). No significant differences in viral shedding were observed between co-infected and virus-only mice, at days 3 and 6 post-HSV-2 infection (Figure 2C), which suggests that the increased pathology following a prior Nb infection was not a result of changes to viral replication. Histological analysis of vaginal tissue at day 6 post-HSV-2 infection revealed increased vaginal epithelial ulceration in co-infected mice when compared with HSV-2-only mice (Figure 2D). IF analysis of vaginal tissue at day 3 post-HSV-2 infection identified a trend for increased epithelial cell necrosis (i.e., loss of membranous Bcat, dispersed DNA, but negative for apoptosis marker c-Casp-3) at the site of ulcer formation in co-infected mice when compared with HSV-2-only mice (Figures 2E and S4). This supports virus-induced epithelial necrosis, rather than apoptosis, underlying epithelial ulceration and that prior Nb infection enhances the onset of necrosis.

Further analysis of vaginal epithelial cells at day 3 post-HSV-2 infection identified reduced expression of MHCI on Nb + HSV-2 vaginal epithelial cells and a trend for less MHCII (Figure 2F). Suppressed MHC presentation in virally infected epithelial cells is associated with evasion of host cytotoxic lymphocyte responses (Neumann et al., 2003; Orr et al., 2005). Host interferon responses can counteract virus-induced downregulation of MHC expression (Härle et al., 2001; Mikloska and Cunningham, 2001; Parr and Parr, 1999). We found reduced vaginal epithelial MHC expression in co-infected mice associated with reduced

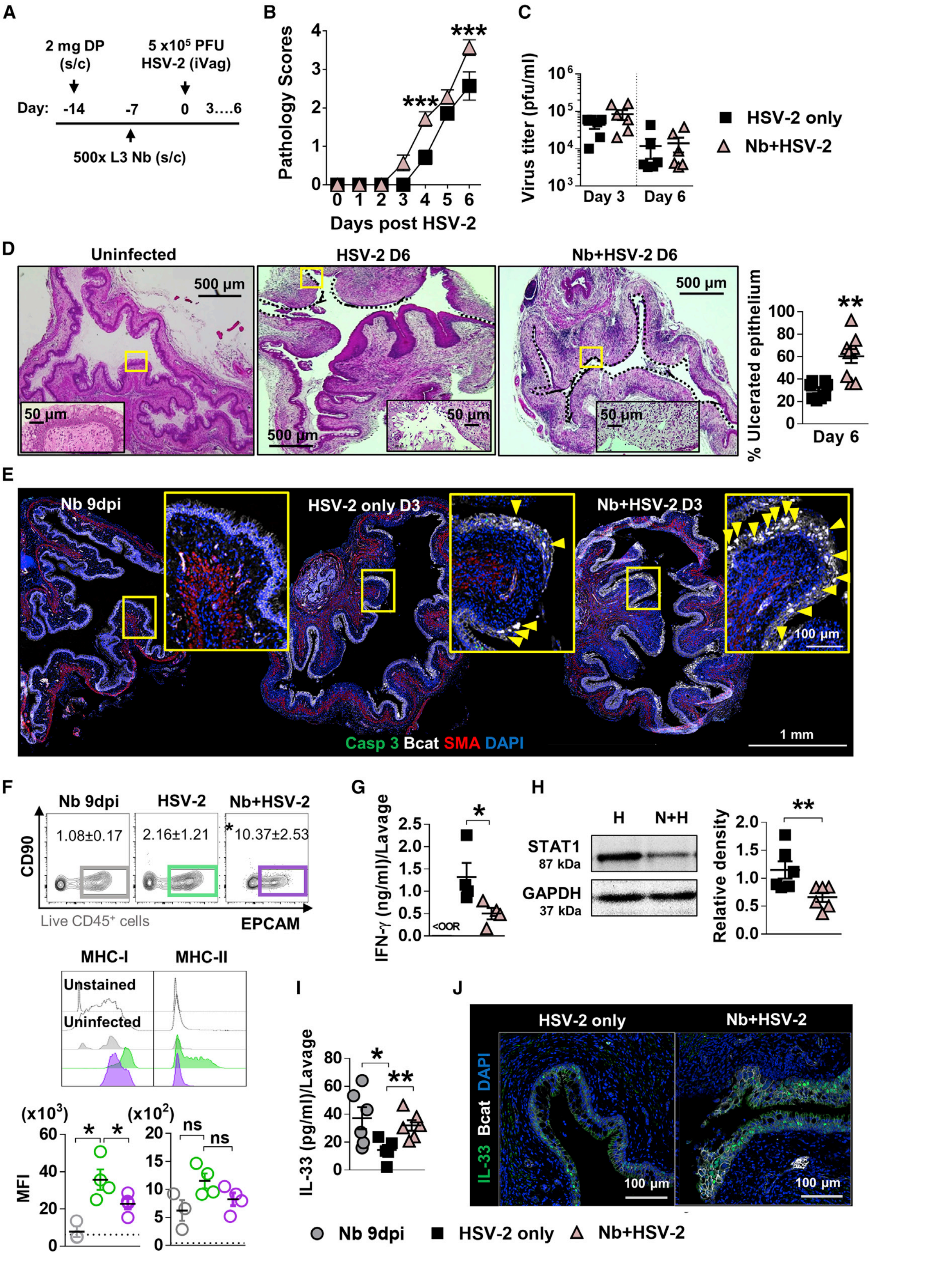

Figure 2. Prior Nb exposure results in earlier and exacerbated HSV-2 pathology

(A) 7 days post-Nb infection, mice were infected intravaginally with 5×10^5 plaque-forming units (PFUs) HSV-2.

(B) Viral progression was determined by daily pathology scoring.

(legend continued on next page)

CellPress
OPEN ACCESS

detection of antiviral IFN-γ (Figure 2G) in vaginal lavages compared with HSV-2-only mice at day 2 post-viral infection. Levels of IFN-γ-induced transcription factor STAT1 were also reduced in the FGT of co-infected mice compared with virus-only controls (Figure 2H). Together, these findings indicate that prior Nb infection may impair epithelial anti-viral responses; however, this effect may be insufficient to alter viral load. We also detected raised IL-33 levels in lavages of co-infected mice compared with HSV-2-only-infected mice (Figure 2I). IF analysis of HSV-2- and Nb + HSV-2-infected vaginal tissue identified a predominant detection of IL-33 in epithelial cells (Figure 2J). We suggest that the raised IL-33 detected in vaginal lavages is a consequence of increased epithelial necrosis and subsequent release of IL-33 in co-infected mice.

Exacerbated viral pathology is associated with Nb-induced type 2 immunity in the FGT

Histological analysis of FGT at the onset of more severe pathology in Nb + HSV-2 mice (day 3 post-virus infection), identified increased cellular infiltration at the sites of epithelial ulceration in co-infected mice compared with HSV-2 alone (Figure S5A). Flow cytometric analysis of the FGT at this time point supported these observations with significantly increased proportions and numbers of eosinophils, as well as inflammatory monocytes and neutrophils in the FGT of co-infected mice compared with virus-only controls. (Figures 3A and S5B). Importantly, increased FGT eosinophils were a feature of co-infected mice and not HSV-2 infection alone. Numbers of neutrophils, Ly6C^{hi} monocytes, macrophages (CD11b^{+}F4/80^{+}), and eosinophils were also raised in the iLN of Nb + HSV-2 mice (Figure S5C). Sirius red staining confirmed increased infiltration of eosinophils into the genital submucosa compared with virus-only-infected mice at day 3 post-HSV-2 infection (Figures 3B and 3Ci). Eosinophil infiltration was also observed at the vaginal epithelial layer in co-infected tissues but not HSV-2-only-infected tissue (Figure 3Cii). Increased detection of the eosinophil granule protein, major basic protein (MBP) was found in co-infected FGT compared with HSV-2 alone, at day 2 post-virus infection (Figure 3D). This supported a role for eosinophils in causing epithelial necrosis as MBP has been demonstrated to be an important cause of epithelial necrosis (Filley et al., 1982).

To further identify if helminth-induced genital eosinophils could promote pathology following HSV-2 infection, expression of established markers of eosinophil function were quantified (Figures 3E and S5D). This analysis revealed increased expression of Ly6C, a proinflammation marker (Percopo et al., 2017), and integrin CD49d, a marker of eosinophil recruitment (Walsh et al., 1996) and survival (Meerschaert et al., 1999) in Nb + HSV-2 genital eosinophils compared with Nb 9dpi (Figure 3E). Together with increased MBP, this suggests raised persistence and inflammatory action of Nb-induced FGT eosinophils during vaginal viral infection that may promote epithelial pathology.

During Nb infection, epithelial IL-33 has been shown to be a significant contributor to the induction of mucosal type 2 immunity, activating ILC2s, which in turn release IL-5 to promote eosinophil recruitment (Hung et al., 2013; Nussbaum et al., 2013). Here, we identified that Nb infection promoted this effect in the FGT; increased levels of lavage IL-5, along with raised proportions and numbers of Lin^{-}IL-7Rα^{+}ICOS^{+}ST2^{+} ILC2s, and IL-5-producing Lin^{-}IL-7Rα^{+}ICOS^{+} cells were found in the FGT and iLN of Nb + HSV-2 compared with HSV-2-only-infected mice (Figures 3F–3H and S5C). Significantly increased proportions and numbers of IL-5-producing inflammatory monocytes were also found in co-infected mice (Figure 3I). Together, these findings identify that host lymphoid and myeloid IL-5 responses in the FGT provide an environment conducive to enabling eosinophil population expansion in Nb + HSV-2 mice.

To test whether IL-5 contributed to Nb-exacerbated viral pathology, we depleted the cytokine with anti-IL-5 (α-IL-5), prior to and during early HSV-2 infection (Figure S6A). α-IL-5-treated co-infected mice displayed significantly reduced pathology and ulcerated epithelium (Figures 3J and S6B) compared with isotype control. Flow cytometry analysis of iLN and Sirius red staining of vaginal tissue at day 6 post-HSV-2 infection confirmed eosinophil depletion following α-IL-5 treatment (Figures 3J and S6C). Exacerbated viral pathology in helminth-exposed mice was therefore associated with type 2 immune imprinting in uncolonized FGT, which was characterized by an eosinophil influx associated with expanded ILC2 and monocyte sources of IL-5.

Nb-induced raised vaginal HSV-2 pathology is independent of Il4ra signaling

Increased genital pathology in Nb + HSV-2-infected mice correlated with raised canonical type 2 IL-4 and IL-5 responses in the FGT and an impaired IFN-γ response. To identify whether, in addition to IL-5, IL-4/IL-13 also contributed to enhanced pathology, either directly or via a classical type 2 antagonism of type 1

(C) Viral shedding (PFU/mL) was measured by plaque assay of day 3 and 6 vaginal washes.
(D) At day 6 post-HSV-2 infection, vaginal tissue was analyzed by H&E staining. Representative images (n = 4) were taken at ×50 and ×400 magnification. Magnified areas are indicated by yellow boxes. HSV-2 ulcerated epithelium is indicated by black dotted lines and qualified as percentage (%) of ulcerated epithelium.
(E) At day 3 post-HSV-2 infection, vaginal tissue (n = 4) was analyzed by immunofluorescent (IF) staining for β-catenin (Bcat; white), α-smooth muscle actin (SMA; red), hoechst 33342 (blue), and c-Casp-3 (green). Yellow boxes identify magnified areas. Yellow arrowheads identify "necrotic" cells i.e., large Bcat-filled nuclei that are c-Casp-3 negative.
(F) At day 3 post-viral infection, vaginal epithelial cells were isolated and analyzed by flow cytometry (CD45^{-}CD90^{-}EPCAM^{+}): MFI of MHCI and MHCII on vaginal epithelial cells from virus-only and co-infected mice. Dotted line represents the MFI of uninfected epithelial cells.
(G) Levels of IFN-γ in vaginal lavages at day 2 post-HSV-2 infection, determined by ELISA.
(H) At day 2 post-viral infection, levels of STAT1 (87 kDa) and GAPDH (37 kDa) were determined in HSV-2-only (H) and co-infected (N + H) FGT homogenates, by western blot. Density of STAT1 was measured relative to GAPDH.
(I and J) (I) Vaginal IL-33 measured by ELISA (day 2) and (J) IF staining of vaginal tissue (day 3; n = 4). Data are representative of two independent experiments with 4–6 mice per group (mean ± SEM). Statistical significance was calculated by two-way analysis of variance (ANOVA) with Bonferroni correction for multiple comparisons and Mann-Whitney t test. *$p \leq 0.05$, **$p \leq 0.01$, **$p \leq 0.01$, ***$p \leq 0.001$, ns, not significant.

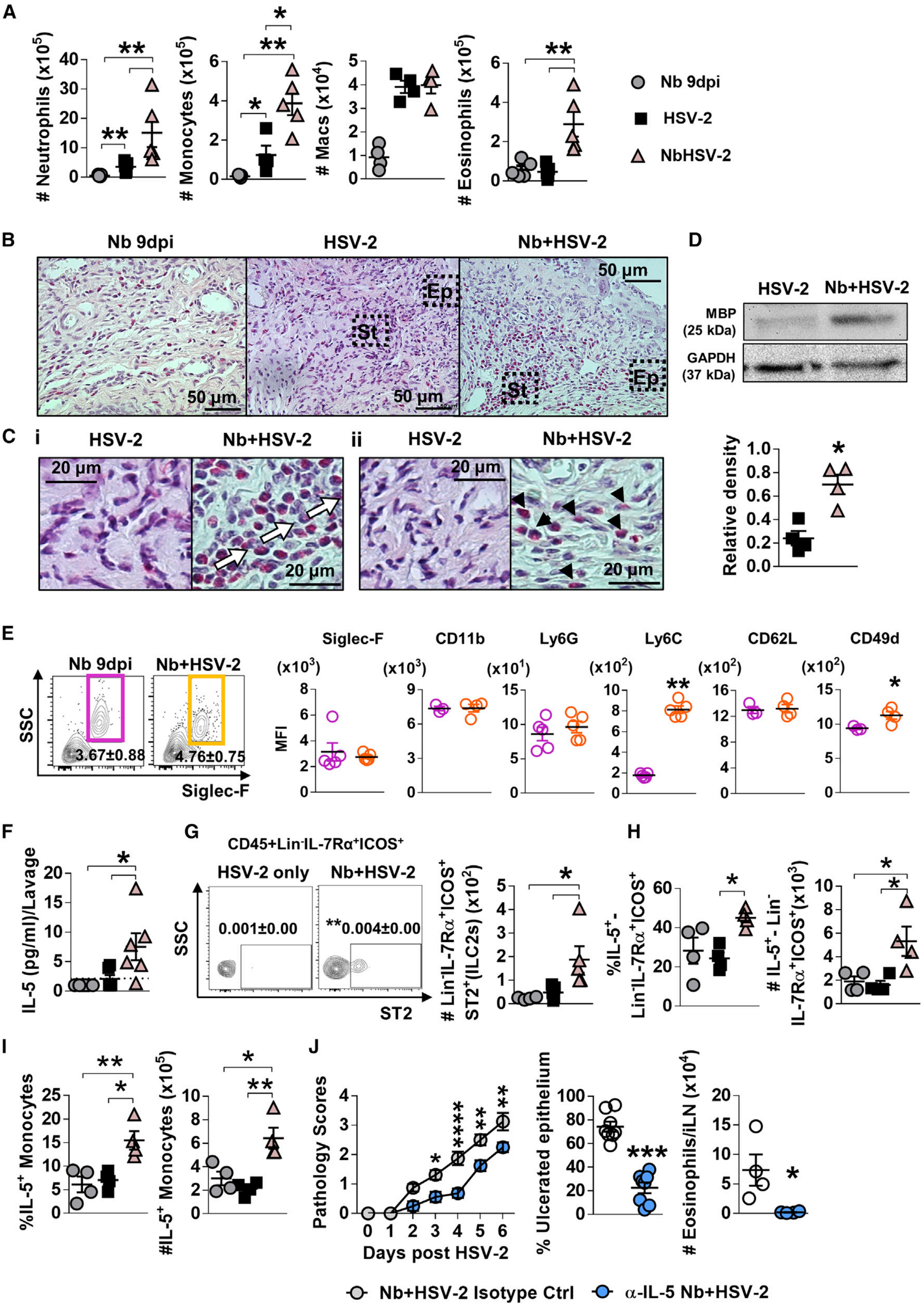

(legend on next page)

CellPress
OPEN ACCESS

immunity, i.e., IL-4 and STAT6 impairment of IFN-γ production (Reese et al., 2014), *Il4ra* knockout (*Il4ra*$^{-/-}$), mice were infected with Nb + HSV-2 or HSV-2 alone. Significantly reduced pathology and viral shedding was observed in HSV-2-infected *Il4ra*$^{-/-}$ mice when compared with wild-type (WT) counterparts. However, unexpectedly, we did not observe significant differences in pathology or viral PFUs, between WT and Il4ra$^{-/-}$ co-infected mice (Figures 4A and 4B). Therefore, Nb-promoted HSV-2 pathology was *Il4ra* independent.

Associated with the onset of genital pathology in both WT and *Il4ra*$^{-/-}$ Nb + HSV-2 co-infected mice was increased IL-5 and reduced IFN-γ levels, in FGT tissue and lavages, respectively, at day 2 post-HSV-2 infection (Figure 4C). Furthermore, histological analysis of vaginal tissue at day 6 post-HSV-2 revealed increased vaginal epithelial ulceration in WT and *Il4ra*$^{-/-}$ co-infected mice compared with HSV-2-only controls (Figure 4D). Sirius red staining exposed increased infiltration of eosinophils in the vaginal stroma and epithelium of co-infected WT and *Il4ra*$^{-/-}$ mice (Figure 4E). Flow cytometry analysis confirmed that heightened HSV-2 pathology following Nb infection was accompanied by raised FGT eosinophils in both WT and *Il4ra*$^{-/-}$ co-infected mice at day 3 post-viral infection (Figure 4F). Of note was the detection of raised eosinophil populations in *Il4ra*$^{-/-}$ co-infected mice. We suggest that this may be a consequence of persistence of Nb infection in these mice. Together, these findings and those in Figure 3 identify that enhanced pathology in co-infected mice is independent of *Il4ra* and instead mediated by the activation of an IL-5 type 2 immune axis.

Il4ra-independent, IL-33-mediated induction of FGT eosinophils

To test whether epithelial-damage-associated IL-33 release could contribute to eosinophil recruitment in the FGT, we challenged WT and Il4ra$^{-/-}$ mice intravaginally with the serine protease papain (Figure 5A), which promotes IL-33 release predominantly by vaginal epithelial cells (Oh et al., 2017) and has been shown to drive an IL-33-dependent abrogation of host control of HSV-2 (Oh et al., 2016). We found that papain treatment resulted in equivalent raised numbers of FGT eosinophils in both WT and *Il4ra*$^{-/-}$ mice compared with untreated controls (Figure 5B). Moreover, induction of FGT ILC2s following intravaginal papain treatment, was also maintained in the absence of *Il4ra* signaling (Figure 5C).

To identify if IL-33 contributed to raised pathology in co-infection, we treated Nb + HSV-2 mice intravaginally with the IL-33 inhibitor heligmosomoides polygyrus alarmin release inhibitor (HpARI) (Figure 5D) (Osbourn et al., 2017). Both WT and *Il4ra*$^{-/-}$ HpARI-treated co-infected mice displayed reduced pathology compared with untreated counterparts (Figure 5E), as well as reduced epithelial ulceration at day 6 post-HSV-2 infection (Figure 5F). Flow cytometry analysis at this time point showed a trend for reduced numbers of ILC2s and significantly less IL-5-producing ILC2s in the FGT of HpARI-treated Nb + HSV-2 mice compared with untreated counterparts (Figures S7B and S7C). Together, these findings support IL-33 driving an *Il4ra*-independent induction of the IL-5 type 2 immune axis, which contributes to exacerbated HSV-2 pathology in co-infected mice.

Eosinophil depletion abrogates helminth-exacerbated HSV-2 pathology

To confirm if Nb-induced FGT eosinophils contributes to elevated HSV-2 pathology, we depleted eosinophils using α-Siglec-F antibody prior to virus infection (Figure 6A). α-Siglec-F treatment significantly reduced eosinophils in the FGT, iLN, spleen and lung compared with treatment with isotype control (Figure S7D). Following HSV-2 infection, α-Siglec-F-treated co-infected mice displayed rescued pathology equivalent to HSV-2-only mice and significantly less than isotype-treated co-infected mice (Figure 6B). No significant differences in viral shedding at day 3 and day 6 post-HSV-2 infection were found (Figure 6C). These findings were also supported by co-infection of ΔdblGata1$^{-/-}$ mice, which lack eosinophils, also resulting in significant reduction in vaginal pathology (Figure S7E). Histological analysis of vaginal tissue at day 6 post-HSV-2 infection demonstrated reduced ulcerated epithelium in α-Siglec-F-treated Nb + HSV-2 mice equivalent to that seen in virus-only-infected mice (Figure 6D). IF analysis of vaginal tissue at this time point showed equivalent detection of c-Casp-3-positive cells in the intact epithelium of all groups, notable loss of vaginal epithelium (Bcat) was observed in isotype-treated Nb + HSV-2 mice compared with HSV-2-only and α-Siglec-F-treated co-infected mice (Figure 6E). Flow cytometry analysis and Sirius red staining

Figure 3. Prior Nb exposure results in elevated inflammation, eosinophil infiltration, and ILC2 presence in genital tissue, following HSV-2 vaginal infection

(A) At day 9 post-Nb infection (Nb only) and day 3 post-HSV-2 infection (HSV-2 only and Nb + HSV-2), numbers of FGT myeloid cells were analyzed by flow cytometry.

(B) Vaginal tissue was analyzed by Sirius red staining. Representative images (n = 4) of virus-induced ulcers were taken at ×400 magnification.

(C) Black boxes indicate magnified sections of (Ci) vaginal stroma (St) and (Cii) epithelium (Ep), taken at ×1,000 magnification. White arrows indicate eosinophilic cell infiltration and migration in ulcerated vaginal tissue. Black arrowheads indicate eosinophil presence in vaginal epithelial layer.

(D) At day 2 post-HSV-2 infection, levels of MBP (25 kDa) and GAPDH (37 kDa) were measured in HSV-2-only and Nb + HSV-2 FGT homogenates. Density of MBP was measured relative to that of GAPDH.

(E) MFI of Siglec-F, CD11b, Ly6G, Ly6C, CD62L, and CD49d on FGT eosinophils in Nb 9 dpi and Nb + HSV-2 mice.

(F) Levels of IL-5 in vaginal lavages at day 2 post-virus infection, determined by Luminex. Dotted line represents LLOQ.

(G) Frequencies (mean ± SEM) and numbers of Lin^{-}IL-7Rα^{+}ICOS^{+}ST2^{+} cells (ILC2s) in the FGT of Nb 9 dpi, HSV-2-only and Nb + HSV-2 mice.

(H and I) Frequency and number of (H) Lin^{-}IL-7Rα^{+}ICOS^{+} cells and (I) Ly6C^{hi} monocytes that are IL-5+ in the FGT, determined by flow cytometry. Data are representative of two independent experiments with 5–6 mice per group (mean ± SEM).

(J) Nb-infected mice were treated with 20 μg α-IL-5 or isotype control, on day −2, 0, and 2 post-HSV-2 infection. Viral progression was determined by daily pathology scoring and HSV-2 ulcerated epithelium was qualified as percentage (%) of ulcerated epithelium. At day 6 post-HSV-2 infection, numbers of eosinophils in the iLN were determined by flow cytometry. Data are representative of two independent experiments with 4 mice per group (mean ± SEM). Statistical significance was calculated by two-way ANOVA with Bonferroni correction for multiple comparisons and Mann-Whitney t test. *$p \leq 0.05$, **$p \leq 0.01$, ***$p \leq 0.001$, ****$p \leq 0.0001$.

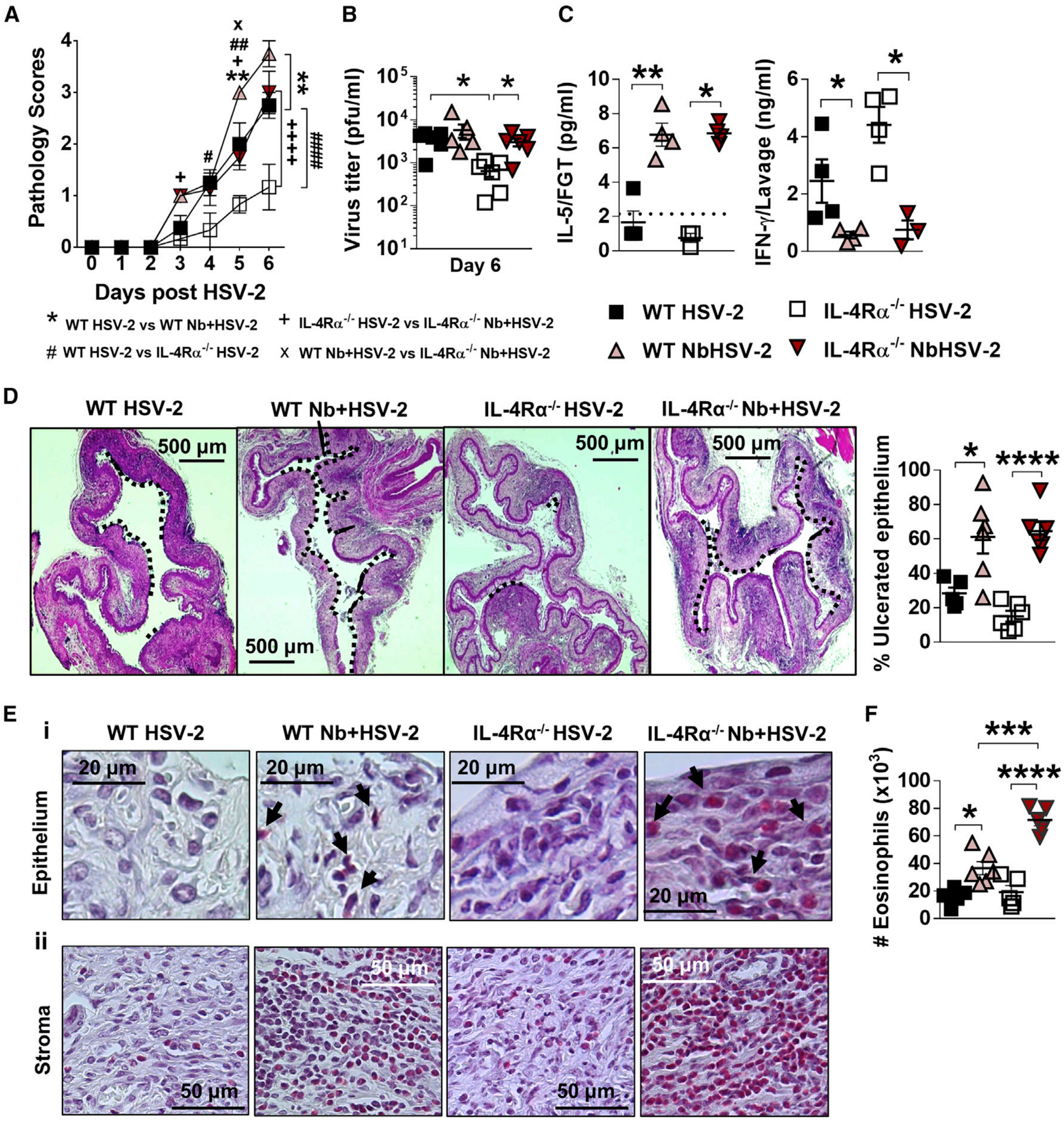

Figure 4. Nb-exacerbated HSV-2 pathology and FGT eosinophil infiltration is *Il4ra* independent

WT and *Il4ra*$^{-/-}$ mice were infected with HSV-2 following Nb exposure as previously described.

(A) HSV-2 progression was determined by daily pathology scoring.

(B) Viral shedding was measured by plaque assay of day 6 vaginal washes.

(C) Genital levels of IL-5 and IFN-γ at day 2 post-HSV-2 infection, determined by Luminex and ELISA, respectively. Dotted line represents LLOQ of Luminex analysis. At 6 dpi, vaginal tissue was analyzed by H&E staining.

(D) Representative sections (n = 3–4), displaying ulceration and inflammation of vaginal tissue. Images were taken at ×50 magnification. HSV-2-ulcerated vaginal epithelium is indicated by black dotted lines and qualified as percentage (%) of ulcerated epithelium.

(E) Representative Sirius-red-stained sections (n = 3–4) of virus-induced (Ei) epithelial ulcers and (Eii) stromal inflammation. Black arrows indicate eosinophil presence in the vaginal epithelial layer. Images were taken at ×400 and ×1,000 magnification.

(F) Numbers (×10^3) of FGT eosinophils in WT and *Il4ra*$^{-/-}$ co-infected mice compared with HSV-2-only controls. Data are representative of two independent experiments with 3–6 mice per group (mean ± SEM). Statistical significance was calculated by two-way ANOVA with Bonferroni correction for multiple comparisons. *p ≤ 0.05, **p ≤ 0.01, ***p ≤ 0.001, ****p ≤ 0.0001.

of the FGT at days 3 and 6, respectively, confirmed a significant reduction in FGT eosinophils in α-Siglec-F-treated co-infected mice (Figures 6F and 6G). These findings support increased epithelial ulceration and reduced tissue integrity in co-infected mice, as being mediated by FGT eosinophil accumulation. Along with these findings, α-Siglec-F treatment also reduced numbers

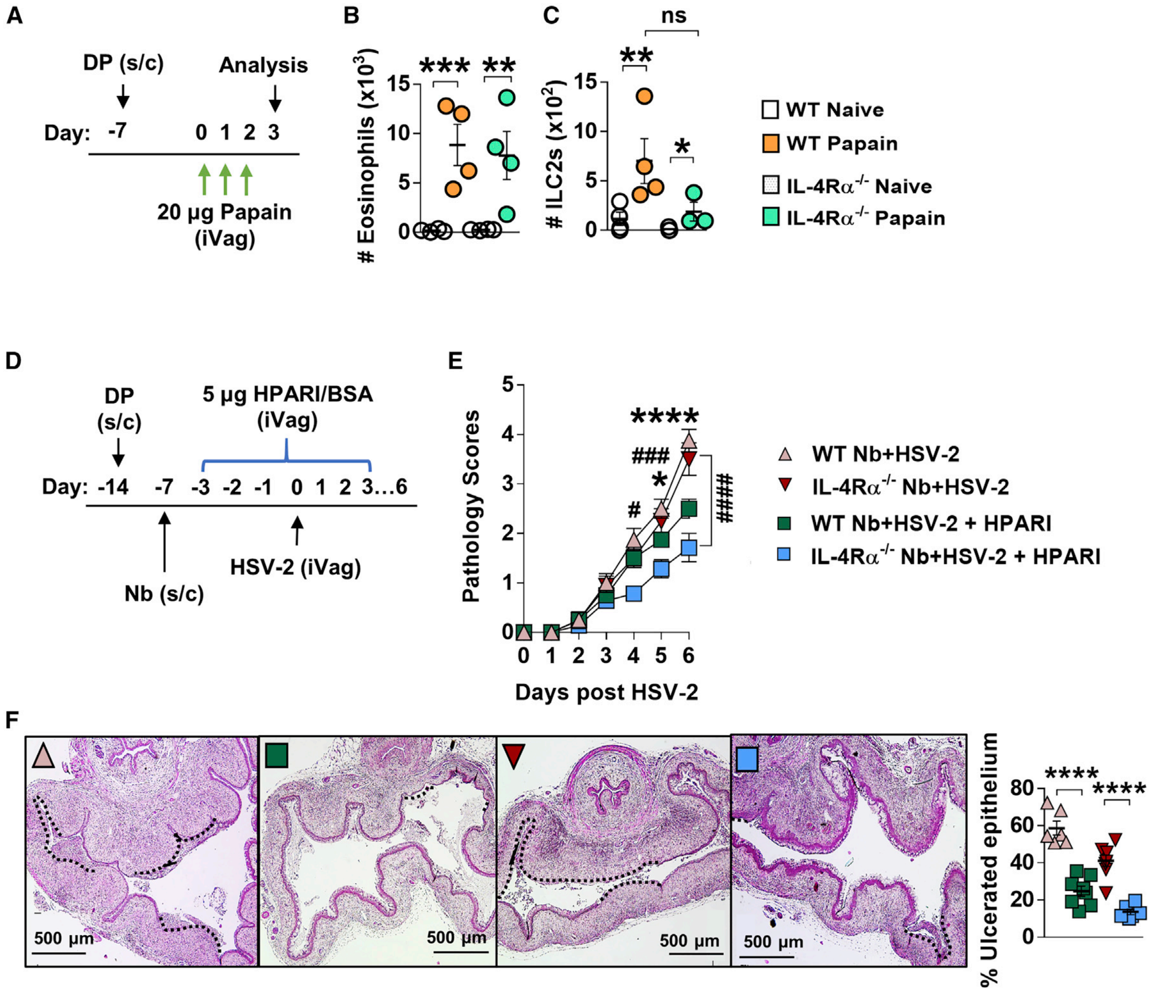

Figure 5. Epithelial IL-33-induced FGT eosinophil inflammation in the absence of *Il4ra* signaling

(A) WT and *Il4ra*$^{-/-}$ mice were treated intravaginally with 20 µg papain for 3 days. The next day, FGT cells were analyzed by flow cytometry.

(B and C) Numbers of FGT (B) eosinophils (x10^3) and (C) ILC2s (×10^2) in papain-treated and untreated WT and *Il4ra*$^{-/-}$ mice. Data are representative of two experiments with 3–4 mice per group (mean ± SEM).

(D) To inhibit vaginal IL-33, co-infected mice were treated intravaginally with helminth-derived HpARI (day −3 to 3 post-HSV-2).

(E) HSV-2 progression in WT and *Il4ra*$^{-/-}$ co-infected HpARI-treated mice and BSA-treated controls was determined by daily pathology scoring (*WT Nb + HSV-2 versus WT Nb + HSV-2 + HpARI, #*Il4ra*$^{-/-}$ Nb + HSV-2 versus *Il4ra*$^{-/-}$ Nb + HSV-2 + HpARI).

(F) Representative H&E-stained sections (n = 4) of ulcerated vaginal tissue. Images were taken at ×50 magnification. HSV-2-ulcerated vaginal epithelium is indicated by black dotted lines and qualified as percentage (%) of ulcerated epithelium. Data are representative of two independent experiments with 4–6 mice per group. Statistical significance was calculated by two-way ANOVA with Bonferroni correction for multiple comparisons and Mann-Whitney t test. *p ≤ 0.05, **p ≤ 0.01, ***p ≤ 0.001, ****p ≤ 0.0001, ns, not significant.

of ILC2s in FGT (Figure 6H). This reduction in FGT ILC2s associated with significantly reduced levels of vaginal IL-33 in α-Siglec-F-treated co-infected mice (Figure 6I). Together, these data demonstrate that the exacerbation of HSV-2 pathology following prior Nb infection is dependent on an Nb-induced FGT eosinophil recruitment and bystander tissue damage.

DISCUSSION

In this study, we identify that systemic immunity to nematode infection results in a type 2 immune profile in uncolonized female genital tissue, a salient feature of this is an enhanced and persistent FGT eosinophil population. Additionally, we show that prior Nb infection results in a step increase in pathology to a subsequent vaginal HSV-2 infection. Depletion of eosinophils protected against this increased HSV-2-induced genital pathology. Furthermore, increased HSV-2 ulceration was dependent on IL-5 and IL-33 and correlated with an *Il4ra*-independent induction of ILC2s, which would further promote eosinophil action in the FGT. These findings may provide a mechanistic framework that explains the association between raised type 2 cytokine profiles in cervical fluids of nematode-infected women and their increased risk of viral infection (Gravitt et al., 2016).

Diverse roles of IL-33 during viral infections have been identified. For example, IL-33 receptor ST2 signaling drives type 2 immune pathology during respiratory viral infection

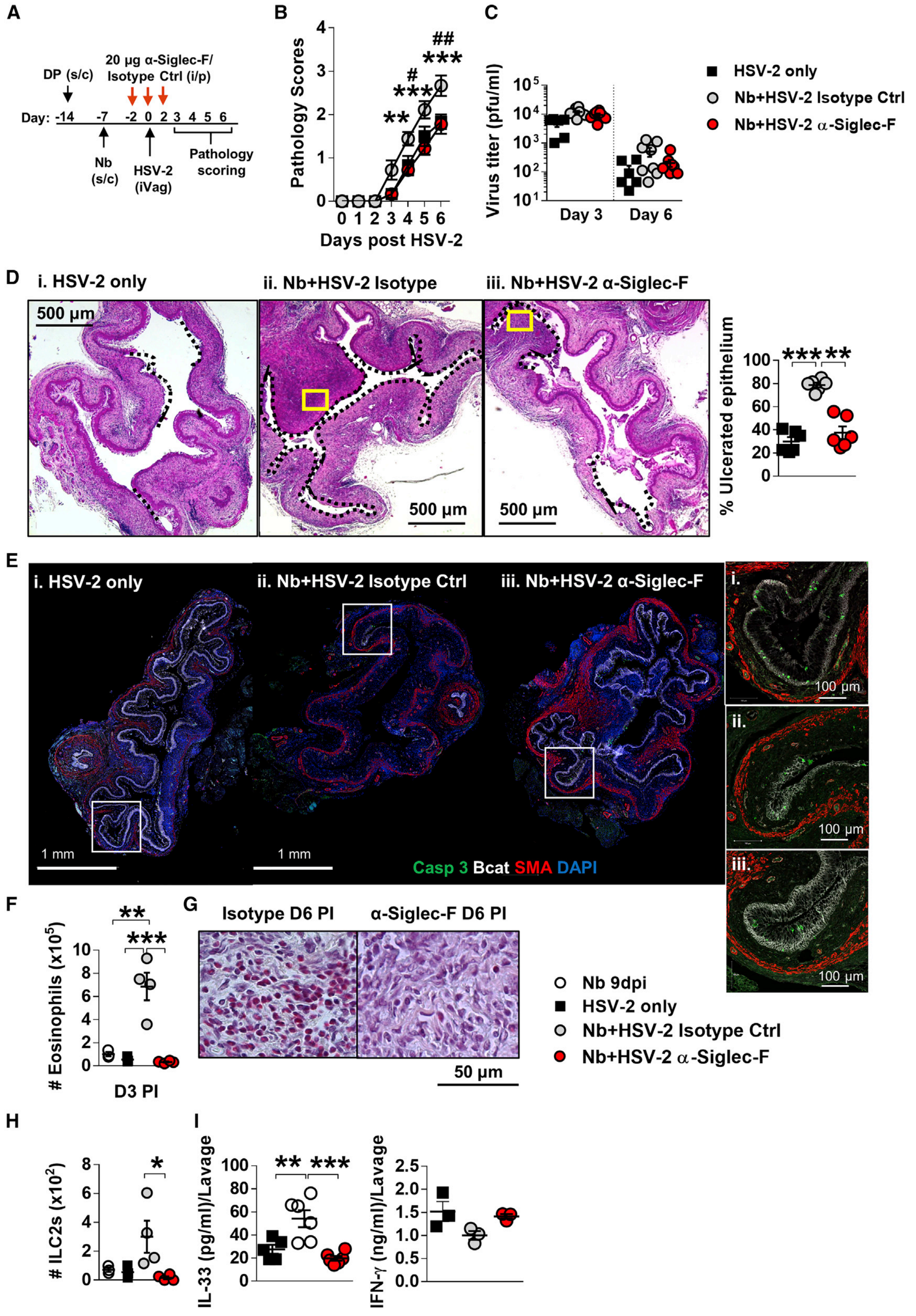

(legend on next page)

(Walzl et al., 2001); IL-33 mediates influenza airway pathology by eliciting IL-13 production by innate lymphoid cells (Chang et al., 2011). Conversely, IL-33 has shown to enhance cytotoxic and memory T cell responses to virus infection (Bonilla et al., 2012) and vaccine challenge (McLaren et al., 2019).

In the vagina, Oh et al. (2016) demonstrated adverse effects of IL-33 on adaptive T cells responses and viral control. Here, we identify an IL-33-promoted type 2 immune environment in the FGT following helminth exposure, which enhances innate immune pathology during subsequent HSV-2 infection. In agreement with others, we also identify the epithelial barrier as the predominant source of IL-33 in the vagina (Oh et al., 2016; Pichery et al., 2012). Our demonstration of eosinophil and ILC2 FGT infiltration, in response to established papain-driven induction of epithelial IL-33 release, further supports these findings. Moreover, we also identified that local inhibition of IL-33 at the epithelial barrier reduced helminth-exacerbated HSV-2 pathology. This body of work supports vaginal epithelial cells to be the key source of IL-33-mediated type 2 immunity in the FGT during co-infection. Recent studies have identified myeloid sources of IL-33 to play roles in downregulating mucosal inflammation (Hung et al., 2020a, 2020b; Jackson et al., 2020; Sell et al., 2020). The findings we present here do not currently suggest a role for cells other than epithelial cells as a contributing source of IL-33. Moreover, our findings support a pro-inflammatory role for IL-33 in the presented co-infection scenario.

Our identification that pathology was a result of increased eosinophil numbers in the FGT was anatomically unexpected, considering the protective role eosinophils have shown during upper FGT damage caused by *Chlamydia* infection (Vicetti Miguel et al., 2017). But our findings do agree with roles of helminth-induced eosinophils in parasite-colonized tissue causing pathology. Expanded eosinophil numbers following parasite colonization or transit of host tissue are well established as a contributor to pathologies resulting from helminth migration including pulmonary eosinophilia, Loeffler's syndrome (Akuthota and Weller, 2012; Chitkara and Krishna, 2006; Ekin et al., 2016; Simmons et al., 2019), and eosinophilic meningitis following *Angiostrongylus cantonensis* infection (McBride et al., 2017). However, the influence of helminth elicited eosinophils on host ability to control unrelated infections is not well understood. Demonstrations of strong associations between eosinophil numbers and mycobacterial and hookworm infections suggest that the eosinophil contribution to bystander immunity is likely to be significant (O'Shea et al., 2018). The findings presented in this study identify key players and mediators required to understand the importance of nematode-induced type 2 immune networks in controlling inflammation and tissue integrity in mucosal-based tissues. This supports future consideration of eosinophils in the FGT as potential drivers of pathology caused by STVIs.

This study also supports Nb infection induction of eosinophils in the FGT as the basis by which pathology to HSV-2 pathology is promoted. Our data support Nb infection conditioning of the FGT via a cycle of eosinophil recruitment promoting IL-33 release, which support expanded ILC2 and monocytes populations that generate increased IL-5 levels and therefore increased promotion of eosinophils in the FGT, thereby driving an environment that elevates tissue pathology following an HSV-2 infection (Figure 7). That this immune environment is appropriate for maintaining FGT eosinophil populations is supported by our demonstration of maintained eosinophil accumulation 12 days post-parasite expulsion. Bystander tissue damage and inflammation in the FGT following Nb infection, despite no direct parasite colonization is possibly directly mediated by eosinophil degranulation as described by others (Galioto et al., 2006; Knott et al., 2009, 2007; Patnode et al., 2014). Eosinophil granule MBP is an established cause of epithelial necrosis (Filley et al., 1982; Kato et al., 2012), and our demonstration of this being increased in our co-infection model suggests such a mechanism underlies increased pathology.

An unexpected feature of our study was that the eosinophil-driven response and raised pathology was largely independent of *Il4ra* expression. *Il4ra* expression is widely demonstrated as a pre-requisite for optimal mucosal eosinophil responses. Studies addressing eosinophil expansion and responses in allergic airway inflammation (Cohn et al., 1999; Nieuwenhuizen et al., 2012), respiratory viral infection (Castilow et al., 2008; Johnson et al., 2003), and helminth infection (Mearns et al., 2008; Spencer et al., 2001) all show noticeably reduced eosinophil responses in *Il4ra*$^{-/-}$ mice. However, IL-5 is accepted as also being the critical type 2 driver of eosinophil development, activation, and survival (Coffman et al., 1989; Daly et al., 1999; Dent et al., 1999; Herndon and Kayes, 1992). In support of our findings, others have demonstrated IL-33 induction of T cell IL-5 independently of IL-4, and STAT-6 being sufficient to induce eosinophils in IL-4$^{-/-}$ mice has been reported (Kurowska-Stolarska et al., 2008). Our demonstration of papain induced IL-33 rapidly generating an *Il4ra*-independent vaginal ILC2 expansion

Figure 6. Depletion of eosinophils rescues HSV-2 pathology in co-infected mice

(A) Co-infected mice were treated with 20 μg α-Siglec-F or isotype control antibody at days 5, 7, and 9 post-Nb infection.

(B) Viral progression in HSV-2-only, α-Siglec-F-, and isotype-treated mice was determined by daily pathology scoring (*Nb + HSV-2 isotype control versus Nb + HSV-2 α-Siglec-F; #HSV-2 only versus Nb + HSV-2 isotype control).

(C) Viral shedding was measured by plaque assay of days 3 and 6 vaginal washes.

(D) Representative H&E-stained sections (n = 3) of vaginal tissue at day 6 post-HSV-2 infection. Images were taken at ×50 magnification. Ulcerated vaginal epithelium is indicated by black dotted lines and qualified as percentage (%) of ulcerated epithelium. Yellow boxes indicate magnified sections in (G).

(E) IF analysis of day 6 post-HSV-2 vaginal tissue. White boxes indicate magnified sections.

(F) Numbers of FGT eosinophils at day 3 post-virus infection in isotype control and α-Siglec-F-treated Nb + HSV-2 mice compared with Nb 9dpi and HSV-2 only controls.

(G) Representative magnified sections (n = 3) of Sirius-red-stained vaginal tissue at day 6 post-HSV-2. Images were taken at ×1,000 magnification.

(H) Numbers of FGT ILC2s at day 3 post-HSV-2 infection.

(I) At day 2 post-HSV-2 infection, lavage or FGT levels of IL-33 and IFN-γ were measured by ELISA. Data are representative of two independent experiments with 3 mice per group (mean ± SEM). Statistical significance was calculated by two-way ANOVA with Bonferroni correction for multiple comparisons and Mann-Whitney t test. *$p \leq 0.05$, **$p \leq 0.01$, ***$p \leq 0.001$.

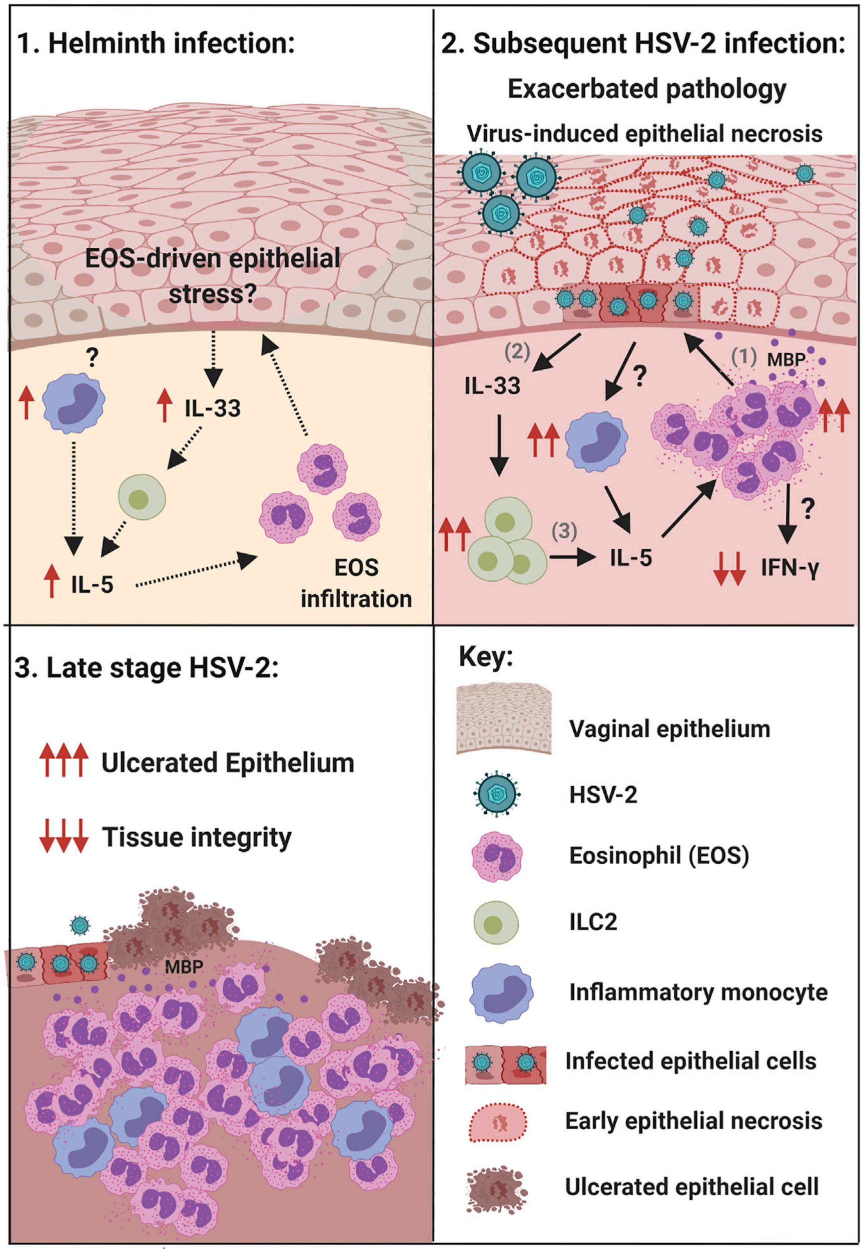

Figure 7. Helminth-induced FGT eosinophils mediate exacerbated vaginal pathology during subsequent lytic viral infection

(A) We hypothesize that Nb-induced infiltration of eosinophils and inflammatory monocytes in uncolonized FGT, results in epithelial stress and release of epithelial "alarmin" IL-33, which supports the local activation of ILC2 and release of IL-5, essential for eosinophil survival.

(B) The consequence of this during a subsequent virus infection was exacerbated pathology caused by virus-induced epithelial necrosis. Type 2 immunity and inflammation is amplified following HSV-2 infection: (1) eosinophil release of granule proteins promotes epithelial necrosis and (2) further release of IL-33, which (3) expands ILC2s that are a source of IL-5, along with infiltrating monocytes. Impaired anti-viral IFN-γ responses associated with eosinophil accumulation in the FGT.

(C) During late-stage disease, epithelial ulceration is increased, and tissue integrity is lost. Created with BioRender.com.

and eosinophil accumulation equivalent to that seen in WT mice identifies redundancy in *Il4ra* signaling in our model. Rapid induction of both ILC2 and eosinophils, suggests T cell independence and lack of *Il4ra* largely precludes a role for IL-13. Our observations and experiments therefore strongly support an IL-33, ILC2/monocyte, IL-5 axis driving vaginal eosinophils.

The current understanding of eosinophil function in the FGT is limited, yet the evidence indicates complex functions, depending on the location of the eosinophil response. Related to our findings, antibiotic-mediated vaginal dysbiosis has been shown to increase HSV-2 vaginal pathology, via epithelial IL-33 impairment of anti-viral immunity; however, this study does not address any role for eosinophils in promoting pathology (Oh et al., 2016). Conversely, eosinophils in the uterus have been shown to promote endometrial repair and reduce tissue destruction following *Chlamydia trachomatis* murine infection (Vicetti Miguel et al., 2017). Our results clearly demonstrate that nematode-induced genital eosinophil infiltration mediates vaginal tissue disruption, predisposing an exacerbation of HSV-2-induced ulceration in the vagina.

In the co-infection model presented here, helminth infection is naturally cleared by an immune-competent host. In Il4ra$^{-/-}$ mice, Nb infections persist (Horsnell et al., 2013; Urban et al., 1998), which suggests that our findings will also have relevance to chronic helminth infections. Chronic enteric helminth infection has been shown to systemically alter immunity in the lung mucosa, protecting against pulmonary virus infection (McFarlane et al., 2017). Irrespective, further investigation is needed to understand the systemic effects of chronic helminth infections on genital susceptibility to viral infections.

In conclusion, we have shown that an acute, self-resolving nematode infection systemically induced canonical type 2 immunity in uncolonized genital tissue. Moreover, helminth-induced genital eosinophils were associated with vaginal tissue disruption, elevated IL-33 responses, and expansion of genital ILC2s and IL-5 levels during subsequent HSV-2 infection. This directly demonstrates systemic influences of gastrointestinal nematode infections on genital responses to viral infection and provides important experimental support for the relevance of reported clinical associations between soil-transmitted helminth infection and vaginal viral infection (Gravitt et al., 2016). These findings represent a conceptual advance in our understanding of how a non-FGT

CellPress
OPEN ACCESS

infection can systemically alter pathogenesis to an important STVI.

STAR★METHODS

Detailed methods are provided in the online version of this paper and include the following:

- KEY RESOURCES TABLE
- RESOURCE AVAILABILITY
  - Lead contact
  - Materials availability
  - Data and code availability
- EXPERIMENTAL MODELS AND SUBJECT DETAILS
  - Cells
  - Virus
  - Animals
  - Parasite maintenance and infection
- METHODS DETAILS
  - Intravaginal infection with HSV-2
  - Intravaginal papain treatment
  - Intravaginal HpARI treatment
  - Antibody depletions
  - Histology
  - Flow cytometry
  - Enzyme-linked immunosorbent assay (ELISA) and luminex
  - Western blotting
- QUANTIFICATION AND STATISTICAL ANALYSIS

SUPPLEMENTAL INFORMATION

Supplemental Information can be found online at https://doi.org/10.1016/j.chom.2021.02.004.

ACKNOWLEDGMENTS

This study was supported by German Research Foundation (DFG) within the "German-Africa Cooperation Projects in Infectiology" (grant LA 2746/2); CNRS of Orléans (France) and European funding in Region Centre-Val de Loire (FEDER no 2016-00110366 BIO-TARGET and EX005756 BIO-TARGET II); and the Poliomyelitis Research Foundation (PRF; 17/08). A.C., W.G.C.H., L.E.L., A.F., M.R., and L.M. were supported by the DFG; A.C. was supported by the National Research Foundation of South Africa (NRF, grant 120685). P.M.V., M.M.-A., and M.J.O. were supported by the Research Council of Norway (Centre of Excellence (223255/F50), and Young Research Talent (274760). B.G.D. is a senior research associate of the F.R.S.-FNRS, Belgium. This research was funded in part by the Wellcome Trust (grant: 203135/Z/16/Z). For the purpose of open access, the author has applied a CC BY public copyright licence to any Author Accepted Manuscript version arising from this submission.

AUTHOR CONTRIBUTIONS

A.C. and M.G.D. performed experiments and the parasite lifecycle. P.M.V., M.M.-A., and A.F. performed experiments and analyzed data. W.G.C.H., A.C., M.R., B.R., M.J.O., and A.F.C. designed the experiments, analyzed the data, prepared the figures, and wrote the paper. F.B. supervised mouse breeding and provided Il4ra$^{-/-}$ mice. W.G.C.H. and L.E.L. acquired funding and supervised the work. H.J.M., M.J.O., B.R., and B.G.D. provided reagents. All authors discussed the results and commented on the paper.

DECLARATION OF INTERESTS

The authors declare no conflict of interest.

Received: June 22, 2020
Revised: December 8, 2020
Accepted: February 8, 2021
Published: March 12, 2021

REFERENCES

Akuthota, P., and Weller, P.F. (2012). Eosinophilic pneumonias. Clin. Microbiol. Rev. *25*, 649–660.

Blackwell, A.D., Tamayo, M.A., Beheim, B., Trumble, B.C., Stieglitz, J., Hooper, P.L., Martin, M., Kaplan, H., and Gurven, M. (2015). Helminth infection, fecundity, and age of first pregnancy in women. Science *350*, 970–972.

Blaho, J.A., Morton, E.R., and Yedowitz, J.C. (2005). Herpes simplex virus: propagation, quantification, and storage. Curr. Protoc. Microbiol. 14E.1.1–14E.1.23.

Bobat, S., Darby, M., Mrdjen, D., Cook, C., Logan, E., Auret, J., Jones, E., Schnoeller, C., Flores-Langarica, A., Ross, E.A., et al. (2014). Natural and vaccine-mediated immunity to Salmonella Typhimurium is impaired by the helminth Nippostrongylus brasiliensis. PLoS Negl. Trop. Dis. *8*, e3341.

Bonilla, W.V., Fröhlich, A., Senn, K., Kallert, S., Fernandez, M., Johnson, S., Kreutzfeldt, M., Hegazy, A.N., Schrick, C., Fallon, P.G., et al. (2012). The alarmin interleukin-33 drives protective antiviral CD8+ T cell responses. Science *335*, 984–989.

Borchers, M.T., Crosby, J., Farmer, S., Sypek, J., Ansay, T., Lee, N.A., and Lee, J.J. (2001). Blockade of CD49d inhibits allergic airway pathologies independent of effects on leukocyte recruitment. Am. J. Physiol. Lung Cell. Mol. Physiol. *280*, L813–L821.

Castilow, E.M., Meyerholz, D.K., and Varga, S.M. (2008). IL-13 is required for eosinophil entry into the lung during respiratory syncytial virus vaccine-enhanced disease. J. Immunol. *180*, 2376–2384.

Chang, S., and Aune, T.M. (2007). Dynamic changes in histone-methylation 'marks' across the locus encoding interferon-gamma during the differentiation of T helper type 2 cells. Nat. Immunol. *8*, 723–731.

Chang, Y.J., Kim, H.Y., Albacker, L.A., Baumgarth, N., McKenzie, A.N.J., Smith, D.E., Dekruyff, R.H., and Umetsu, D.T. (2011). Innate lymphoid cells mediate influenza-induced airway hyper-reactivity independently of adaptive immunity. Nat. Immunol. *12*, 631–638.

Chetty, A., Omondi, M.A., Butters, C., Smith, K.A., Katawa, G., Ritter, M., Layland, L., and Horsnell, W. (2020). Impact of helminth infections on female reproductive health and associated diseases. Front. Immunol. *11*, 577516.

Chitkara, R.K., and Krishna, G. (2006). Parasitic pulmonary eosinophilia. Semin. Respir. Crit. Care Med. *27*, 171–184.

Coffman, R.L., Seymour, B.W., Hudak, S., Jackson, J., and Rennick, D. (1989). Antibody to interleukin-5 inhibits helminth-induced eosinophilia in mice. Science *245*, 308–310.

Cohn, L., Homer, R.J., MacLeod, H., Mohrs, M., Brombacher, F., and Bottomly, K. (1999). Th2-induced airway mucus production is dependent on IL-4Ralpha, but not on eosinophils. J. Immunol. *162*, 6178–6183.

Daly, C.M., Mayrhofer, G., and Dent, L.A. (1999). Trapping and immobilization of Nippostrongylus brasiliensis larvae at the site of inoculation in primary infections of interleukin-5 transgenic mice. Infect. Immun. *67*, 5315–5323.

Dent, L.A., Daly, C.M., Mayrhofer, G., Zimmerman, T., Hallett, A., Bignold, L.P., Creaney, J., and Parsons, J.C. (1999). Interleukin-5 transgenic mice show enhanced resistance to primary infections with Nippostrongylus brasiliensis but not primary infections with Toxocara canis. Infect. Immun. *67*, 989–993.

Ekin, S., Sertogullarindan, B., Gunbatar, H., Arisoy, A., and Yildiz, H. (2016). Loeffler's syndrome: an interesting case report. Clin. Respir. J. *10*, 112–114.

Filley, W.V., Holley, K.E., Kephart, G.M., and Gleich, G.J. (1982). Identification by immunofluorescence of eosinophil granule major basic protein in lung tissues of patients with bronchial asthma. Lancet *2*, 11–16.

Freeman, E.E., Weiss, H.A., Glynn, J.R., Cross, P.L., Whitworth, J.A., and Hayes, R.J. (2006). Herpes simplex virus 2 infection increases HIV acquisition in men and women: systematic review and meta-analysis of longitudinal studies. AIDS *20*, 73–83.

Galioto, A.M., Hess, J.A., Nolan, T.J., Schad, G.A., Lee, J.J., and Abraham, D. (2006). Role of eosinophils and neutrophils in innate and adaptive protective immunity to larval Strongyloides stercoralis in mice. Infect. Immun. *74*, 5730–5738.

Gravitt, P.E., Marks, M., Kosek, M., Huang, C., Cabrera, L., Olortegui, M.P., Medrano, A.M., Trigoso, D.R., Qureshi, S., Bardales, G.S., et al. (2016). Soil-transmitted helminth infections are associated With an increase in human papillomavirus prevalence and a T-helper type 2 cytokine signature in cervical fluids. J. Infect. Dis. *213*, 723–730.

Grayson, M.H., Van der Vieren, M., Sterbinsky, S.A., Michael Gallatin, W., Hoffman, P.A., Staunton, D.E., and Bochner, B.S. (1998). alphadbeta2 integrin is expressed on human eosinophils and functions as an alternative ligand for vascular cell adhesion molecule 1 (VCAM-1). J. Exp. Med. *188*, 2187–2191.

Härle, P., Noisakran, S., and Carr, D.J.J. (2001). The application of a plasmid DNA encoding IFN-α1 postinfection enhances cumulative survival of herpes simplex virus Type 2 vaginally infected mice. J. Immunol. *166*, 1803–1812.

Henderson, W.R., Jr., Chi, E.Y., Albert, R.K., Chu, S.J., Lamm, W.J., Rochon, Y., Jonas, M., Christie, P.E., and Harlan, J.M. (1997). Blockade of CD49d (alpha4 integrin) on intrapulmonary but not circulating leukocytes inhibits airway inflammation and hyperresponsiveness in a mouse model of asthma. J. Clin. Invest. *100*, 3083–3092.

Herndon, F.J., and Kayes, S.G. (1992). Depletion of eosinophils by anti-IL-5 monoclonal antibody treatment of mice infected with Trichinella spiralis does not alter parasite burden or immunologic resistance to reinfection. J. Immunol. *149*, 3642–3647.

Horsnell, W.G., Cutler, A.J., Hoving, J.C., Mearns, H., Myburgh, E., Arendse, B., Finkelman, F.D., Owens, G.K., Erle, D., and Brombacher, F. (2007). Delayed goblet cell hyperplasia, acetylcholine receptor expression, and worm expulsion in SMC-specific IL-4Ralpha-deficient mice. PLoS Pathog. *3*, e1.

Horsnell, W.G.C., Darby, M.G., Hoving, J.C., Nieuwenhuizen, N., McSorley, H.J., Ndlovu, H., Bobat, S., Kimberg, M., Kirstein, F., Cutler, A.J., et al. (2013). IL-4Rα-associated antigen processing by B cells promotes immunity in Nippostrongylus brasiliensis infection. PLoS Pathog. *9*, e1003662.

Hung, L.Y., Lewkowich, I.P., Dawson, L.A., Downey, J., Yang, Y., Smith, D.E., and Herbert, D.R. (2013). IL-33 drives biphasic IL-13 production for noncanonical type 2 immunity against hookworms. Proc. Natl. Acad. Sci. USA *110*, 282–287.

Hung, L.-Y., Pastore, C.F., Douglas, B., and Herbert, D.R. (2020a). Myeloid-derived interleukin-33 limits the severity of dextran sulfate sodium (DSS)-induced colitis. Am. J. Pathol. *191*, 30505–30508.

Hung, L.-Y., Tanaka, Y., Herbine, K., Pastore, C., Singh, B., Ferguson, A., Vora, N., Douglas, B., Zullo, K., Behrens, E.M., et al. (2020b). Cellular context of IL-33 expression dictates impact on anti-helminth immunity. Sci. Immunol. *5*, eabc6259.

Jackson, S.R., Costa, M.F.D.M., Pastore, C.F., Zhao, G., Weiner, A.I., Adams, S., Palashikar, G., Quansah, K., Hankenson, K., Herbert, D.R., et al. (2020). R-spondin 2 mediates neutrophil egress into the alveolar space through increased lung permeability. BMC Res. Notes *13*, 54.

Johnson, T.R., Parker, R.A., Johnson, J.E., and Graham, B.S. (2003). IL-13 is sufficient for respiratory syncytial virus G glycoprotein-induced eosinophilia After respiratory syncytial virus challenge. J. Immunol. *170*, 2037–2045.

Kato, M., Ishioka, T., Kita, H., Kozawa, K., Hayashi, Y., and Kimura, H. (2012). Eosinophil granular proteins damage bronchial epithelial cells infected with respiratory syncytial virus. Int. Arch. Allergy Immunol. *158* (suppl 1), 11–18.

Knott, M.L., Matthaei, K.I., Foster, P.S., and Dent, L.A. (2009). The roles of eotaxin and the STAT6 signalling pathway in eosinophil recruitment and host resistance to the nematodes Nippostrongylus brasiliensis and Heligmosomoides bakeri. Mol. Immunol. *46*, 2714–2722.

Knott, M.L., Matthaei, K.I., Giacomin, P.R., Wang, H., Foster, P.S., and Dent, L.A. (2007). Impaired resistance in early secondary Nippostrongylus brasiliensis infections in mice with defective eosinophilopoeisis. Int. J. Parasitol. *37*, 1367–1378.

Kurowska-Stolarska, M., Kewin, P., Murphy, G., Russo, R.C., Stolarski, B., Garcia, C.C., Komai-Koma, M., Pitman, N., Li, Y., Niedbala, W., et al. (2008). IL-33 induces antigen-specific IL-5+ T cells and promotes allergic-induced airway inflammation independent of IL-4. J. Immunol. *181*, 4780–4790.

Lee, A.J., Chen, B., Chew, M.V., Barra, N.G., Shenouda, M.M., Nham, T., van Rooijen, N., Jordana, M., Mossman, K.L., Schreiber, R.D., et al. (2017). Inflammatory monocytes require type I interferon receptor signaling to activate NK cells via IL-18 during a mucosal viral infection. J. Exp. Med. *214*, 1153–1167.

Looker, K.J., Elmes, J.A.R., Gottlieb, S.L., Schiffer, J.T., Vickerman, P., Turner, K.M.E., and Boily, M.C. (2017). Effect of HSV-2 infection on subsequent HIV acquisition: an updated systematic review and meta-analysis. Lancet Infect. Dis. *17*, 1303–1316.

Looker, K.J., Magaret, A.S., Turner, K.M.E., Vickerman, P., Gottlieb, S.L., and Newman, L.M. (2015). Global estimates of prevalent and incident herpes simplex virus Type 2 infections in 2012. PLoS One *10*, e114989.

Marshak, J.O., Dong, L., and Koelle, D.M. (2014). The murine intravaginal HSV-2 challenge model for investigation of DNA vaccines. Methods Mol. Biol. *1144*, 305–327.

McBride, A., Chau, T.T.H., Hong, N.T.T., Mai, N.T.H., Anh, N.T., Thanh, T.T., Van, T.T.H., Xuan, L.T., Sieu, T.P.M., Thai, L.H., et al. (2017). Angiostrongylus cantonensis is an important cause of eosinophilic meningitis in Southern Vietnam. Clin. Infect. Dis. *64*, 1784–1787.

McFarlane, A.J., McSorley, H.J., Davidson, D.J., Fitch, P.M., Errington, C., Mackenzie, K.J., Gollwitzer, E.S., Johnston, C.J.C., MacDonald, A.S., Edwards, M.R., et al. (2017). Enteric helminth-induced type I interferon signaling protects against pulmonary virus infection through interaction with the microbiota. J. Allergy Clin. Immunol. *140*, 1068–1078.e6.

McLaren, J.E., Clement, M., Marsden, M., Miners, K.L., Llewellyn-Lacey, S., Grant, E.J., Rubina, A., Gimeno Brias, S., Gostick, E., Stacey, M.A., et al. (2019). IL-33 augments virus-specific memory T cell inflation and potentiates the efficacy of an attenuated Cytomegalovirus-based vaccine. J. Immunol. *202*, 943–955.

Mearns, H., Horsnell, W.G., Hoving, J.C., Dewals, B., Cutler, A.J., Kirstein, F., Myburgh, E., Arendse, B., and Brombacher, F. (2008). Interleukin-4-promoted T helper 2 responses enhance Nippostrongylus brasiliensis-induced pulmonary pathology. Infect. Immun. *76*, 5535–5542.

Meerschaert, J., Vrtis, R.F., Shikama, Y., Sedgwick, J.B., Busse, W.W., and Mosher, D.F. (1999). Engagement of alpha4beta7 integrins by monoclonal antibodies or ligands enhances survival of human eosinophils in vitro. J. Immunol. *163*, 6217–6227.

Mesnil, C., Raulier, S., Paulissen, G., Xiao, X., Birrell, M.A., Pirottin, D., Janss, T., Starkl, P., Ramery, E., Henket, M., et al. (2016). Lung-resident eosinophils represent a distinct regulatory eosinophil subset. J. Clin. Invest. *126*, 3279–3295.

Mikloska, Z., and Cunningham, A.L. (2001). Alpha and gamma interferons inhibit herpes simplex virus Type 1 infection and spread in epidermal cells after axonal transmission. J. Virol. *75*, 11821–11826.

Milligan, G.N., and Bernstein, D.I. (1997). Interferon-γ enhances resolution of herpes simplex virus Type 2 infection of the murine genital tract. Virology *229*, 259–268.

Nakajima, H., Sano, H., Nishimura, T., Yoshida, S., and Iwamoto, I. (1994). Role of vascular cell adhesion molecule 1/very late activation antigen 4 and intercellular adhesion molecule 1/lymphocyte function-associated antigen 1 interactions in antigen-induced eosinophil and T cell recruitment into the tissue. J. Exp. Med. *179*, 1145–1154.

Neumann, J., Eis-Hübinger, A.M., and Koch, N. (2003). Herpes simplex virus Type 1 targets the MHC Class II processing pathway for immune evasion. J. Immunol. *171*, 3075–3083.

Nieuwenhuizen, N.E., Kirstein, F., Jayakumar, J., Emedi, B., Hurdayal, R., Horsnell, W.G.C., Lopata, A.L., and Brombacher, F. (2012). Allergic airway disease is unaffected by the absence of IL-4Rα-dependent alternatively activated macrophages. J. Allergy Clin. Immunol. *130*, 743–750.e8.

CellPress
OPEN ACCESS

Nussbaum, J.C., Van Dyken, S.J., von Moltke, J., Cheng, L.E., Mohapatra, A., Molofsky, A.B., Thornton, E.E., Krummel, M.F., Chawla, A., Liang, H.E., et al. (2013). Type 2 innate lymphoid cells control eosinophil homeostasis. Nature *502*, 245–248.

Oh, J.E., Kim, B.C., Chang, D.H., Kwon, M., Lee, S.Y., Kang, D., Kim, J.Y., Hwang, I., Yu, J.W., Nakae, S., et al. (2016). Dysbiosis-induced IL-33 contributes to impaired antiviral immunity in the genital mucosa. Proc. Natl. Acad. Sci. USA *113*, E762–E771.

Oh, J.E., Oh, D.S., Jung, H.E., and Lee, H.K. (2017). A mechanism for the induction of type 2 immune responses by a protease allergen in the genital tract. Proc. Natl. Acad. Sci. USA *114*, E1188–E1195.

Orr, M.T., Edelmann, K.H., Vieira, J., Corey, L., Raulet, D.H., and Wilson, C.B. (2005). Inhibition of MHC class I is a virulence factor in herpes simplex virus infection of mice. PLoS Pathog. *1*, e7.

Osbourn, M., Soares, D.C., Vacca, F., Cohen, E.S., Scott, I.C., Gregory, W.F., Smyth, D.J., Toivakka, M., Kemter, A.M., le Bihan, T., et al. (2017). HpARI protein secreted by a helminth parasite suppresses interleukin-33. Immunity *47*, 739–751.e5.

O'Shea, M.K., Fletcher, T.E., Muller, J., Tanner, R., Matsumiya, M., Bailey, J.W., Jones, J., Smith, S.G., Koh, G., Horsnell, W.G., et al. (2018). Human hookworm infection enhances mycobacterial growth inhibition and associates with reduced risk of tuberculosis infection. Front. Immunol. *9*, 2893.

Parr, M.B., and Parr, E.L. (1999). The role of gamma interferon in immune resistance to vaginal infection by herpes simplex virus type 2 in mice. Virology *258*, 282–294.

Patnode, M.L., Bando, J.K., Krummel, M.F., Locksley, R.M., and Rosen, S.D. (2014). Leukotriene B4 amplifies eosinophil accumulation in response to nematodes. J. Exp. Med. *211*, 1281–1288.

Percopo, C.M., Brenner, T.A., Ma, M., Kraemer, L.S., Hakeem, R.M., Lee, J.J., and Rosenberg, H.F. (2017). SiglecF+Gr1hi eosinophils are a distinct subpopulation within the lungs of allergen-challenged mice. J. Leukoc. Biol. *101*, 321–328.

Phipps, W., Nakku-Joloba, E., Krantz, E.M., Selke, S., Huang, M.L., Kambugu, F., Orem, J., Casper, C., Corey, L., and Wald, A. (2016). Genital herpes simplex virus Type 2 shedding Among adults With and Without HIV infection in Uganda. J. Infect. Dis. *213*, 439–447.

Pichery, M., Mirey, E., Mercier, P., Lefrancais, E., Dujardin, A., Ortega, N., and Girard, J.P. (2012). Endogenous IL-33 is highly expressed in mouse epithelial barrier tissues, lymphoid organs, brain, embryos, and inflamed tissues: in situ analysis using a novel IL-33–LacZ gene TRAP reporter strain. J. Immunol. *188*, 3488–3495.

Reese, T.A., Wakeman, B.S., Choi, H.S., Hufford, M.M., Huang, S.C., Zhang, X., Buck, M.D., Jezewski, A., Kambal, A., Liu, C.Y., et al. (2014). Helminth infection reactivates latent γ-herpesvirus via cytokine competition at a viral promoter. Science *345*, 573–577.

Rolot, M., Dougall, A.M., Chetty, A., Javaux, J., Chen, T., Xiao, X., Machiels, B., Selkirk, M.E., Maizels, R.M., Hokke, C., et al. (2018). Helminth-induced IL-4 expands bystander memory CD8+ T cells for early control of viral infection. Nat. Commun. *9*, 4516.

Sell, E.A., Ortiz-Carpena, J.F., Herbert, D.R., and Cohen, N.A. (2020). Tuft cells in the pathogenesis of chronic rhinosinusitis with nasal polyps and asthma. Ann. Allergy Asthma Immunol. *126*, 143–151.

Simmons, R.P., Dudzinski, D.M., Shepard, J.-A.O., Hurtado, R.M., and Coffey, K.C. (2019). Case 16–2019: A 53-year-old man with cough and eosinophilia. N. Engl. J. Med. *380*, 2052–2059.

Spencer, L., Shultz, L., and Rajan, T.V. (2001). Interleukin-4 receptor–Stat6 signaling in murine infections with a tissue-dwelling nematode parasite. Infect. Immun. *69*, 7743–7752.

Szabo, S.J., Dighe, A.S., Gubler, U., and Murphy, K.M. (1997). Regulation of the interleukin (IL)-12R beta 2 subunit expression in developing T helper 1 (Th1) and Th2 cells. J. Exp. Med. *185*, 817–824.

Thapa, M., Kuziel, W.A., and Carr, D.J.J. (2007). Susceptibility of CCR5-deficient mice to genital herpes simplex virus type 2 is linked to NK cell mobilization. J. Virol. *81*, 3704–3713.

Thawer, S., Auret, J., Schnoeller, C., Chetty, A., Smith, K., Darby, M., Roberts, L., Mackay, R.M., Whitwell, H.J., Timms, J.F., et al. (2016). Surfactant protein-D is essential for immunity to helminth infection. PLOS Pathog. *12*, e1005461.

Ujma, S., Carse, S., Chetty, A., Horsnell, W., Clark, H., Madsen, J., Mackay, R.M., Watson, A., Griffiths, M., Katz, A.A., et al. (2019). Surfactant protein A impairs genital HPV16 Pseudovirus infection by innate immune cell activation in A murine model. Pathogens *8*, 288.

Urban, J.F., Noben-Trauth, N., Donaldson, D.D., Madden, K.B., Morris, S.C., Collins, M., and Finkelman, F.D. (1998). IL-13, IL-4Ralpha, and Stat6 are required for the expulsion of the gastrointestinal nematode parasite Nippostrongylus brasiliensis. Immunity *8*, 255–264.

Veen, J.C., Grootendorst, D.C., Bel, E.H., Smits, H.H., Van Der Keur, M., Sterk, P.J., and Hiemstra, P.S. (1998). CD11b and L-selectin expression on eosinophils and neutrophils in blood and induced sputum of patients with asthma compared with normal subjects. Clin. Exp. Allergy *28*, 606–615.

Vicetti Miguel, R.D., Quispe Calla, N.E., Dixon, D., Foster, R.A., Gambotto, A., Pavelko, S.D., Hall-Stoodley, L., and Cherpes, T.L. (2017). IL-4–secreting eosinophils promote endometrial stromal cell proliferation and prevent Chlamydia-induced upper genital tract damage. Proc. Natl. Acad. Sci. USA *114*, E6892–E6901.

Walker, C., Rihs, S., Braun, R.K., Betz, S., and Bruijnzeel, P.L. (1993). Increased expression of CD11b and functional changes in eosinophils after migration across endothelial cell monolayers. J. Immunol. *150*, 4061–4071.

Walsh, G.M., Symon, F.A., Lazarovils, A.L., and Wardlaw, A.J. (1996). Integrin alpha 4 beta 7 mediates human eosinophil interaction with MAdCAM-1, VCAM-1 and fibronectin. Immunology *89*, 112–119.

Walzl, G., Matthews, S., Kendall, S., Gutierrez-Ramos, J.C., Coyle, A.J., Openshaw, P.J., and Hussell, T. (2001). Inhibition of T1/ST2 during respiratory syncytial virus infection prevents T helper cell type 2 (Th2)- but not Th1-driven immunopathology. J. Exp. Med. *193*, 785–792.

Wei, L., Vahedi, G., Sun, H.W., Watford, W.T., Takatori, H., Ramos, H.L., Takahashi, H., Liang, J., Gutierrez-Cruz, G., Zang, C., et al. (2010). Discrete roles of STAT4 and STAT6 transcription factors in tuning epigenetic modifications and transcription during T helper cell differentiation. Immunity *32*, 840–851.

WHO. (2002). Prevention and control of schistosomiasis and soil-transmitted helminthiasis, World Health Organization technical report series. p.57 https://www.who.int/intestinal_worms/resources/who_trs_912/en/.

Zwiggelaar, R.T., Lindholm, H.T., Fosslie, M., Terndrup Pedersen, M., Ohta, Y., Díez-Sánchez, A., Martín-Alonso, M., Ostrop, J., Matano, M., Parmar, N., et al. (2020). LSD1 represses a neonatal/reparative gene program in adult intestinal epithelium. Sci. Adv. *6*, eabc0367.

STAR★METHODS

KEY RESOURCES TABLE

REAGENT or RESOURCE	SOURCE	IDENTIFIER
**Antibodies**		
Anti-Siglec-F Monoclonal Rat IgG2A Clone: # 238047 (depletion) Cat# MAB17061	R&D systems	AB_2286029
Monoclonal Rat IgG2A Isotype Control Clone: # 54447 Cat# MAB006	R&D systems	AB_357349
Anti-IL-5 Monoclonal Rat IgG1 Clone: TRFK5 (Functional grade, depletion) Cat# 16-7052-81	eBioscience™, Thermo Scientific™	AB_469212
Monoclonal Rat IgG1 Isotype Control Clone: eBRG1 (Functional grade) Cat# 16-4301-81	Invitrogen™, Thermo Scientific™	AB_470153
Anti-mouse CD16/32, clone: 93 Cat# 101302	BioLegend®	AB_312801
Anti-mouse CD45 Alexa Fluor® 700, clone: 30-F11 Cat# 103128	BioLegend®	AB_493715
Anti-mouse CD11b Brilliant violet (BV) 421™, clone: M1/70 Cat# 101235	BioLegend®	AB_10897942
Anti-mouse F4/80 BV605™, clone: BM8 Cat# 123133	BioLegend®	AB_2562305
Anti-mouse Ly6C FITC, clone: HK1.4 Cat# 128005	BioLegend®	AB_1186134
Anti-mouse Ly6G APC Cy7, clone: 1A8 Cat# 127623	BioLegend®	AB_10645331
Anti-mouse Siglec-F PE, clone: S17007L Cat# 155506	BioLegend®	AB_2750235
Anti-mouse lineage cocktail PE (CD3ε, clone: 145-2C11; Ly-6G/Ly-6C, clone: RB6-8C5; CD11b, clone: M1/70; CD45R/B220, clone: RA3-6B2; TER-119, clone: Ter-119) Cat# 133303	BioLegend®	AB_1595553
Anti-mouse IL-7Rα (CD127) PE Cy7, clone: A7R34 Cat# 135013	BioLegend®	AB_1937266
Anti-mouse ICOS APC, clone: C398.4A Cat# 313510	BioLegend®	AB_416334
Anti-mouse ST2 (IL-33Rα) BV421™, clone: DIH9 Cat# 145309	BioLegend®	AB_2565634
Anti-mouse EPCAM (CD326) APC, clone: G8.8 Cat# 118213	BioLegend®	AB_1134105
Anti-mouse CD90.2 BV605™, clone: 30-H12 Cat# 105343	BioLegend®	AB_2632889
Anti-mouse MHCI (H-2Dd) PE, clone: 34-2-12 Cat# 110607	BioLegend®	AB_313488
Anti-mouse MHCII (I-A/I-E) FITC, clone: M5/114.15.2 Cat# 107605	BioLegend®	AB_313320
Anti-mouse CD62L BV605™, clone MEL-14 Cat# 104437	BioLegend®	AB_11125577
Anti-mouse CD49d Alexa Fluor® 647, clone R1-2 Cat# 103613	BioLegend®	AB_528836
Anti-mouse/human CD11b PerCP, clone M1/70 Cat# 101230	BioLegend®	AB_2129374
Anti-mouse/human IL-5 BV421™, clone TRFK5 Cat# 504311	BioLegend®	AB_2563161
Rabbit polyclonal anti-STAT1 Cat# ab47425	abcam	AB_882708
Rabbit polyclonal anti-Major Basic Protein (MBP)	abcam	Cat# ab187523
Rabbit polyclonal anti-GAPDH Cat# ab9485	abcam	AB_307275
Goat anti-rabbit IgG-HRP Cat# ab205718	abcam	AB_2819160
**Bacterial and virus strains**		
Human herpesvirus 2 (strain G) VR-734™	ATCC®	-
**Biological samples**		
Heligmosomoides polygyrus Alarmin Release Inhibitor (HpARI)	Dr Henry McSorley	N/A
**Chemicals, peptides, and recombinant proteins**		
Depo Provera®	Pfizer	N/A
Papain Product	Sigma-Aldrich®	Cat# P4762
7-aminoactinomycin D (7-AAD) staining solution	BioLegend®	Cat# 420404
Protease inhibitor cocktail	Sigma-Aldrich®	Cat # P8340

(*Continued on next page*)

CellPress
OPEN ACCESS

Continued		
REAGENT or RESOURCE	SOURCE	IDENTIFIER
Phorbol 12-myristate 13-acetate (PMA)	Sigma-Aldrich®	Cat# P1585
Ionomycin calcium salt	Sigma-Aldrich®	Cat# I3909
Brefeldin A (BFA)	Sigma-Aldrich®	Cat# B6542
LIVE/DEAD™ Fixable Aqua Dead Cell Stain Kit	Invitrogen™, Thermo Scientific™	Cat# L34957
LumiGlo® chemiluminescent substrate	KPL	Cat# 54-12-50
Critical commercial assays		
Pierce™ bicinchoninic acid (BCA) assay	Thermo Scientific™	Cat# 23225
ELISA MAX™ Standard Set Mouse IL-4	BioLegend®	Cat# 431101
ELISA MAX™ Standard Set Mouse IFN-γ	BioLegend®	Cat # 430801
Mouse IL-33 DuoSet ELISA	R&D systems	Cat# DY3626
Pierce™ TMB Substrate Kit	Thermo Scientific™	Cat# 34021
Invitrogen™ Cytokine & Chemokine 36-plex mouse kit ProcartaPlex™ Cat# EPX360-26092-901	Thermo Scientific™	AB_2576123
Experimental models: cell lines		
African green monkey kidney (Vero) cells CCL-81™	ATCC®	CVCL_0059
Experimental models: organisms/strains		
Nippostrongylus brasiliensis	University of Cape Town, WGC Horsnell Group, Division of Immunology, IDM.	N/A
Mouse: BALB/c	University of Cape Town, Faculty of Health Sciences, Research Animal Facility (UCT FHSRAF)	N/A
Mouse: *Il4ra*$^{-/-}$	Prof Frank Brombacher	N/A
Mouse: ΔdblGata1$^{-/-}$	University Hospital of Bonn, Institute for Medical Microbiology, Immunology and Parasitology (originally obtained from The Jackson Laboratory (Bar Harbor, ME, USA)	N/A
Software and algorithms		
FlowJo V10	Tree Star	https://www.flowjo.com/
GraphPad Prism V6		https://www.graphpad.com/scientific-software/prism/
Fiji Image J		https://imagej.nih.gov/ij/
BioRender		https://biorender.com/

RESOURCE AVAILABILITY

Lead contact

Further information and requests for resources and reagents should be directed to and will be fulfilled by the Lead Contact, Dr William G. C. Horsnell (wghorsnell@gmail.com).

Materials availability

This study did not generate new unique reagents.

Data and code availability

This study did not generate any unique datasets or code.

EXPERIMENTAL MODELS AND SUBJECT DETAILS

Cells

African green monkey kidney (Vero) cells were obtained from ATCC (ATCC® CCL-81™, Manassas, VA, USA) and cultured in Dulbecco's Modified Eagle Medium (DMEM, Sigma-Aldrich®) supplemented with 10% FCS, 100 U/ml penicillin, 100mg/ml streptomycin and 2mM glutamine, at 37°C, 5% CO_2.

Virus

Human herpesvirus 2 strain G (HSV-2, ATCC® VR-734™) was propagated in Vero cells (Blaho et al., 2005; Marshak et al., 2014). Confluent Vero cells were washed with serum-free media and the HSV-2 inoculum was added, at a multiplicity of infection (MOI) of 0.1. At 2-3 days post-infection, cells and supernatant was collected and viral titres were determined by plaque assay. Briefly, confluent Vero cells were incubated with serial dilutions of viral stock, at 37°C, 5% CO_2, for 2 hours (hrs) to allow for absorption. The inoculum was then replaced with supplemented DMEM and cells were incubated for 2 days, fixed with methanol + 2% H_2O_2 and stained with Giemsa stain (Sigma-Aldrich®) to determine plaque forming units (PFU). Viral aliquots were stored at -80°C until use.

Animals

Mice were bred and housed in specific pathogen-free conditions at the Research Animal Facility, University of Cape Town, South Africa. Food and water were provided ad libitum. All studies carried out are in accordance with ethical protocols 014/027 or 018/002, approved by the Faculty of Health Science Animal Ethics Committee from the University of Cape Town. Mice were randomly sorted into experimental groups. Female wildtype BALB/c, *Il4ra*$^{-/-}$ and ΔdblGata1$^{-/-}$ (BALB/c background) mice, aged 6-8 weeks, were injected subcutaneously with 2 mg Depo Provera® (Pfizer) in sterile phosphate buffered saline (PBS), 7 days prior to infection, to synchronize estrous cycles and facilitate consistent intravaginal viral infection (Marshak et al., 2014).

Parasite maintenance and infection

N. brasiliensis (Nb) was maintained in male Wistar rats (ethics protocol 014/042 or 018/037). Briefly, rats were injected subcutaneously with 5000 x infectious Nb L3.Feces were collected during peak helminth egg production (day 6-8 post infection). Fecal cultures were prepared by placing a feces/charcaol mix on wet raised filter paper. Hatched L3 arvae migrate to the edge of filter paper and are collected by gently washing off with water. Collected L3 larvae were counted under a disecting microscope and resuspended in an appropriate volume for infection. Mice were infected with 500 x Nb L3 larvae subcutaneously delivered in 200 ul of water, 7 days prior to viral infection.

METHODS DETAILS

Intravaginal infection with HSV-2

Mice were anesthetized and inoculated intravaginally with 5 x 10^5 PFU HSV-2. Virus-associated illness severity was determined by pathology scoring: 0 - No pathology observed; 1 - Slight genital/perianal erythema; 2 - Genital/perianal swelling and erythema; 3 - Genital lesions and/or visible weight loss; 4 - Hind limb paralysis and/or purulent lesions; 5 – Premoribund (Marshak et al., 2014). Vaginal lavages were performed by 10x flushing the vaginal vault with 50 μl sterile PBS. This was repeated three times. Viral shedding was quantified by plaque assay as described previously.

Intravaginal papain treatment

Female WT or *Il4ra*$^{-/-}$ mice were treated subcutaneously with 2 mg Depo Provera® to equilibrate hormone levels. One week later, mice were treated with 20 μg of serine protease Papain intravaginally under deep anesthesia, for three consecutive days.

Intravaginal HpARI treatment

Hormone-synchronized female WT and *Il4ra*$^{-/-}$ mice were infected subcutaneously with 500x L3 Nb, one week prior to intravaginal infection with 5 x 10^5 PFU HSV-2. To inhibit vaginal IL-33, mice were treated intravaginally with 5 μg *Heligmosomoides polygyrus* Alarmin Release Inhibitor (HpARI) (Osbourn et al., 2017) consecutively from 3 days prior until 3 days post HSV-2 infection.

Antibody depletions

To deplete eosinophils and IL-5, mice were treated with 20 μg/mouse anti-Siglec-F (α-Siglec-F, monoclonal Rat IgG2A clone: # 238047, R&D systems) or anti-IL-5 (α-IL-5, monoclonal Rat IgG1 clone: TRFK5, eBioscience™) antibody intraperitoneally on day 5, 7 and 9 post Nb infection. Control mice were treated with rat IgG2A or rat IgG1 isotype antibody, respectively.

Histology

Vaginal tissue was excised and fixed in phosphate-buffered formalin solution (Sigma-Aldrich®) overnight (Horsnell et al., 2007). Following paraffin embedding, tissue was cut into 5 μm cross-sections (2 sections per vaginal at varying depths) and stained with hematoxylin and eosin (H&E) to visualize vaginal epithelial integrity and inflammation. Sections were also stained with hematoxylin and Sirius red, to identify eosinophils. All sections were viewed with Zeiss Axioskop Microscope (Zeiss) and images were taken with a color AxioCam HRc and AxioVision 4.7 supporting software. 'Ulcerated epithelium' was defined as the complete loss of the stratified squamous cells from a given length of vaginal epithelium. 'Total epithelial length' was defined as the total length of the tissue-lumen interface. Total and ulcerated epithelium length was measured using ImageJ Software (NIH) and percentage of ulcerated epithelium was calculated as follows:

% ulcerated epithelium = ("Measured length of ulcerated epithelium" / "Total measured epithelial length") x 100.

Immunofluorescent (IF) staining and confocal imaging: Briefly, paraffin embedded sections (4 μm) were subjected to deparaffination in Neo-clear (Sigma-Aldrich®), followed by rehydration and antigen retrieval in Tris-EDTA buffer (pH 9). Slides were blocked (5% normal goat serum, 5% BSA, 0.3% Tx-100, 0.05% Tween 20 in PBS) and then incubated with primary antibodies (anti-βcatenin, Mouse monoclonal Ab, BD Biosciences, 610154; anti-cleaved caspase 3, Rabbit polyclonal Ab, Cell signaling, 9661) overnight at 4°C (humid). Following three washes, slides were then incubated with appropriate secondary antibodies (goat anti-Rabbit IgG Alexa Fluor 488, Invitrogen, A11034; goat anti-Mouse IgG Alexa Fluor 647, Invitrogen, A32728) for 1 hr at room temperature (dark, humid). Following three washes, slides were incubated with anti-smooth muscle actin (SMA)-Cy3 directly labelled antibody (mouse mAb, Sigma-Aldrich® C6198) and Hoechst 33342 for 30 min at RT (dark, humid).

For IL-33 staining, antigen retrieval was performed by boiling slides in citrate buffer (pH 6), followed by blocking (5% BSA, 0.3% Tx-100, 0.05% Tween 20 in PBS). The primary antibodies (anti-IL33, Goat polyclonal, AF3626, R&D Systems; anti-βcatenin) were incubated in 2.5 % BSA, 0.3% Tx-100, 0.05% Tween 20 in PBS. Secondary antibody (Donkey anti-Goat IgG Alexa Fluor Plus 488, Invitrogen, A32814; Donkey anti-Mouse IgG Alexa Fluor Plus 647, Invitrogen, A32787) incubation was performed in the same buffer for 1 h at RT adding Hoechst 33342.

After four washes, slides were mounted in Fluoromount G medium (Thermo Fisher Scientific) and imaged using Zeiss Airyscan confocal microscope, with a 10x and 20x objective. Images were analyzed using Zen black edition software (Zeiss). Maximal intensity projections are shown (Zwiggelaar et al., 2020).

Flow cytometry

Female genital tissue (FGT, excluding ovaries) was removed from individual mice, finely cut, and digested in supplemented DMEM containing 1% HEPES and 20 μg/ml Liberase™ TL (Roche), for 1 hr at 37°C with gentle shaking. Digested tissue was passed through a 70 μm cell strainer and dispersed cells resuspended in fresh supplemented DMEM. Iliac lymph nodes (iLN) were excised and passed through a 40 μm cell strainer. To isolate vaginal epithelial cells, excised vaginal tissue was incubated in 0.25% Trypsin/EDTA, for 1 hr at 37°C with gentle shaking.

Cells were stained with staining buffer (PBS + 0.5% BSA and 2mM Ethylenediaminetetraacetic acid (EDTA)) containing 2% heat-inactivated rat serum, 1 μg anti-mouse CD16/32 antibody (clone: 93, BioLegend) and fluorochrome-conjugated antibodies against cell-surface markers for 20 min (min) at 4°C (dark): CD45 Alexa Fluor® 700 (clone: 30-F11, BioLegend) to define hematopoietic cells; CD11b Brilliant violet (BV) 421™ (clone: M1/70, BioLegend), F4/80 BV605™ (clone: BM8, BioLegend), Ly6C FITC (clone: HK1.4, BioLegend), Ly6G APC Cy7 (clone: 1A8, BioLegend) and Siglec-F PE (clone: S17007L, BioLegend) to identify myeloid cells; lineage cocktail PE (CD3ϵ, clone: 145-2C11; Ly-6G/Ly-6C, clone: RB6-8C5; CD11b, clone: M1/70; CD45R/B220, clone: RA3-6B2; TER-119, clone: Ter-119, BioLegend), IL-7Rα (CD127) PE Cy7 (clone: A7R34, BioLegend), ICOS APC (clone: C398.4A, BioLegend) and ST2 (IL-33Rα) BV421™ (clone: DIH9, BioLegend) to define ILC2s; and EPCAM (CD326) APC (clone: G8.8, BioLegend), CD90.2 BV605™ (clone: 30-H12, BioLegend), MHCI (H-2D^d) PE (clone: 34-2-12, BioLegend) and MHCII (I-A/I-E) FITC (clone: M5/114.15.2, BioLegend) to identify vaginal epithelial cells. 7-aminoactinomycin D (7-AAD) staining was used to identify 'Live' cells.

For intracellular cytokine staining, cells were incubated in complete DMEM, containing 50 ng/ml Phorbol 12-myristate 13-acetate (PMA; Sigma-Aldrich®) and 1 μg/ml Ionomycin (Sigma-Aldrich®) for 3 hrs at 37°C, in the presence of 10 μg/ml brefeldin A (BFA; Sigma-Aldrich®). Following *ex vivo* stimulation, cells were stained for viability (LIVE/DEAD® Fixable Aqua) and surface markers, fixed in 2% Formalin/PBS, and washed in permeabilization buffer (eBioscience™), before incubation with anti-mouse/human IL-5 BV421™ (clone: TRFK5, BioLegend) for 30 min at 4°C (dark).

Samples were acquired on a BD LSR Fortessa flow cytometer (BD Biosciences) and data were analyzed by FlowJo© V10 (Treestar, Ashland, OR). Appropriately stained compensation beads and unstained controls were run to compensate for spectral overlap between fluorochrome emissions.

Enzyme-linked immunosorbent assay (ELISA) and luminex

Isolated FGT was snap frozen in liquid nitrogen and stored at -80°C until use. For cytokine analysis, tissue was homogenized in RIPA lysis buffer containing protease inhibitor cocktail (Sigma-Aldrich®), using a benchtop homogenizer (Kinematica Polytron™ PT 2500E homogenizer). Homogenates were centrifuged at 10 000 rpm for 10 min to isolate the supernatant. Protein concentrations were quantified by bicinchoninic acid (BCA) assay (Pierce™, Thermo Scientific) and all samples were standardized for cytokine analysis by ELISA or Luminex (Thawer et al., 2016).

Quantification of cytokines IL-4 and IFN-γ was performed using ELISA MAX™ Standard kits (BioLegend) following the manufacturer's instructions. IL-33 quantification was performed using Mouse IL-33 Duoset ELISA kit (R&D systems) as per manufacturer's instructions. The plates were developed with TMB microwell peroxidase substrate system (Thermo Fisher Scientific), and the reaction was stopped with 1M H_3PO_4. The plates were read at an absorbance of 450nm (Lm1) and 570 nm (Lm2; background) using a VersaMax microplate reader (Molecular Devices Corporation, CA, U.S.A).

FGT homogenate and vaginal lavage cytokine levels were measured by Luminex xMap technology using the Invitrogen ProcartaPlex™ 36-plex mouse kit (Cat# EPX360-26092-901, Lot # 189084327), as per manufacturers. The Luminex plate was

read using Bio-Rad Bio-Plex® 200 system and analysis software. Luminex was performed once, with samples from two independent experiments. Analyte levels below the lower detection limits were given an arbitrary value of half the Lower Limit of Quantification (LLOQ), defined by the manufacturer.

Western blotting

For detection of proteins of interest in the FGT, 25 μg of tissue homogenates were analyzed by reduced SDS-PAGE and Western blotting according to conventional protocols (Ujma et al., 2019). Primary antibodies used were rabbit polyclonal anti-STAT1 (abcam; ab47425), rabbit polyclonal anti-Major Basic Protein (MBP; abcam; ab187523) and rabbit polyclonal anti-GAPDH loading control (abcam; ab9485). For signal detection, secondary goat anti-rabbit IgG-HRP (abcam; ab205718) was used. All Western blots were visualized using the LumiGlo® chemiluminescent substrate (KPL, Milford, MA, USA), with the Biospectrum™ 500 Imaging System (UltraViolet Products, UVP, Mile End South, SA, Australia). Densitometry analysis were performed using ImageJ software, to determine relative changes in protein expression.

QUANTIFICATION AND STATISTICAL ANALYSIS

All statistical details (statistical tests used, value of n (number of animals), definition of center, and dispersion and precision measures) can be found in the figure legends. Data are represented as group mean and standard error of the mean (mean±sem). Statistical analysis was performed either by analysis of variance (ANOVA) followed by the Bonferroni multiple comparison test or by non-parametric Mann-Whitney test, with a 95% confidence interval. A *p value* $\leq$ 0.05 was considered significant and are indicated by an asterisk (*). Statistical analyses were performed using GraphPad Prism V6.

Cell Host & Microbe, Volume *29*

Supplemental information

Il4ra-independent vaginal eosinophil accumulation following helminth infection exacerbates epithelial ulcerative pathology of HSV-2 infection

Alisha Chetty, Matthew G. Darby, Pia M. Vornewald, Mara Martín-Alonso, Anna Filz, Manuel Ritter, Henry J. McSorley, Lindi Masson, Katherine Smith, Frank Brombacher, Matthew K. O'Shea, Adam F. Cunningham, Bernhard Ryffel, Menno J. Oudhoff, Benjamin G. Dewals, Laura E. Layland, and William G.C. Horsnell

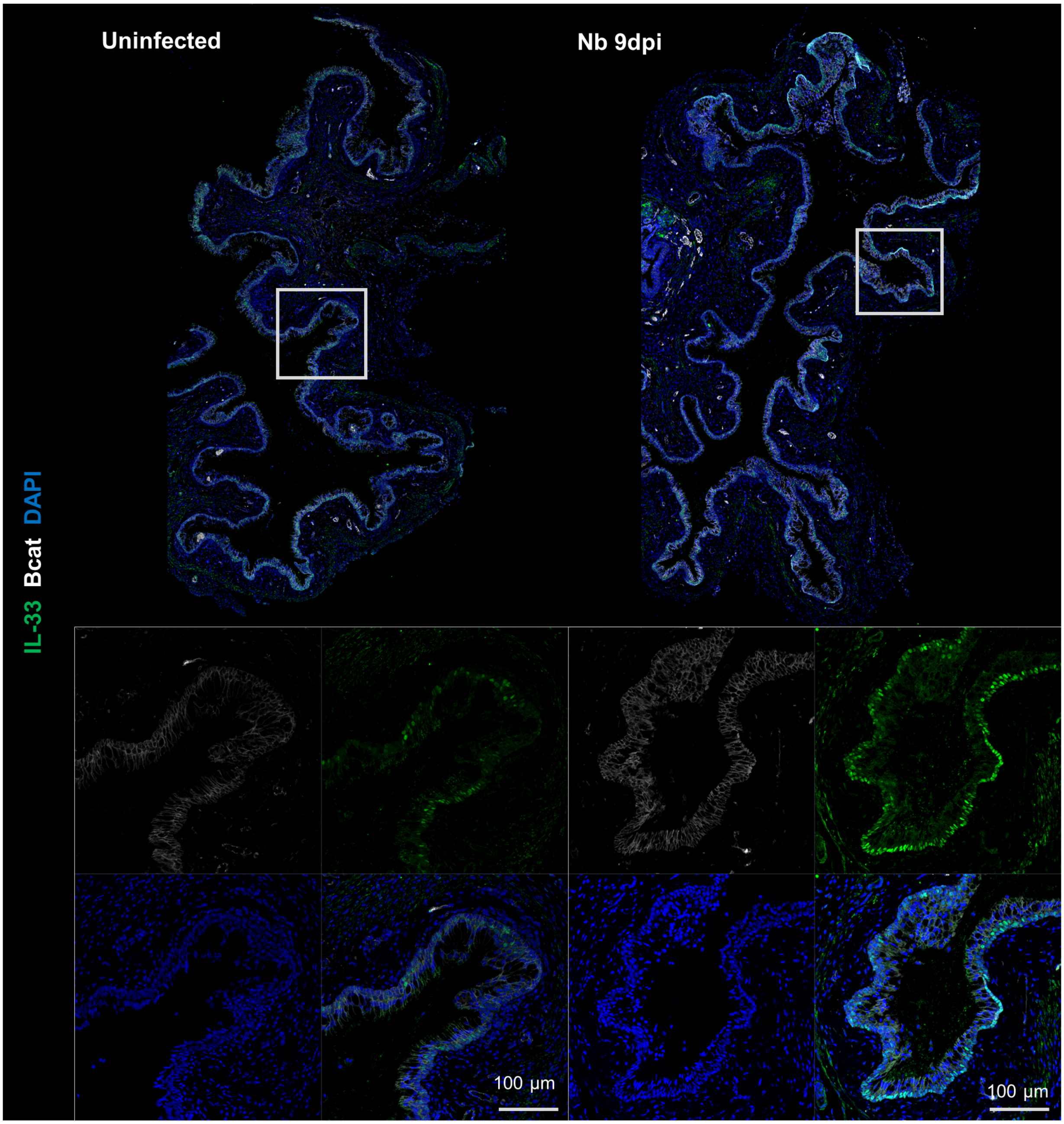

Figure S1. Vaginal epithelial cells are a major source of IL-33, Related to Figure 1: At day 9 post Nb infection (Nb 9dpi), vaginal tissue (n=2) was isolated for immunofluorescent (IF) staining for beta catenin (Bcat; white), IL-33 (green) and Hoechst 33342 (blue). White boxes indicate magnified sections. Data is representative of one experiment with 2 mice per group.

a

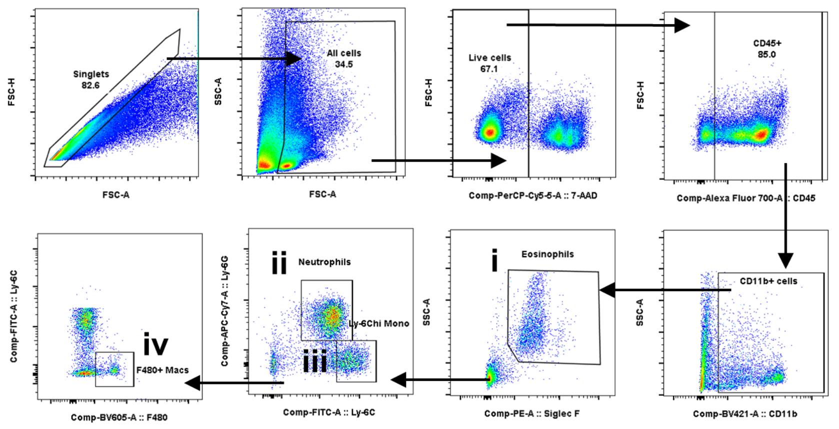

b

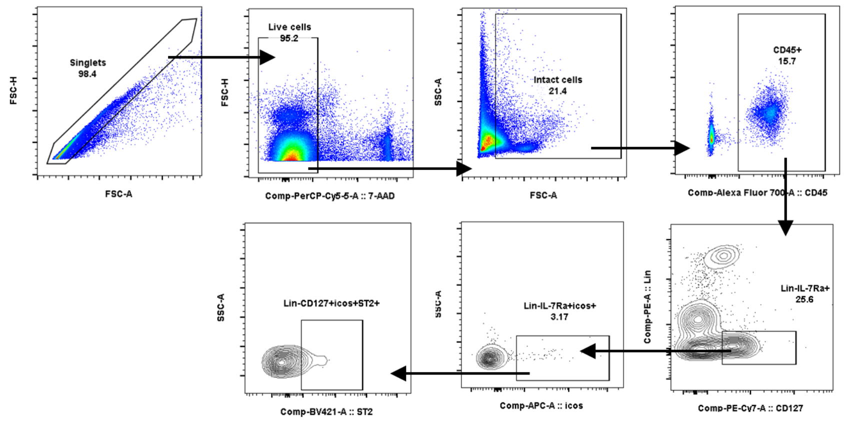

Figure S2. FGT and iLN flow cytometry gating strategy, Related to Star Methods: Flow cytometry: At day 3 post HSV-2 infection. FGT and iLNs were harvested and processed to a single cell suspension. Cells were stained with fluorochrome-conjugated antibodies and analysed by multi-colour flow cytometry. **(a)** The following gating strategy was used to identify **(i)** CD11b^{+}SigLec-F^{+}SSC^{hi} eosinophils, **(ii)** CD11b^{+}Ly-6G^{+} neutrophils, **(iii)** CD11b^{+}Ly-6C^{hi} monocytes and **(iv)** CD11b^{+}F480^{+} macrophages. **(b)** Gating strategy used to identify Lin^{-}IL-7Rα^{+}ICOS^{+}ST2^{+} ILC2s in the FGT and iLN.

a

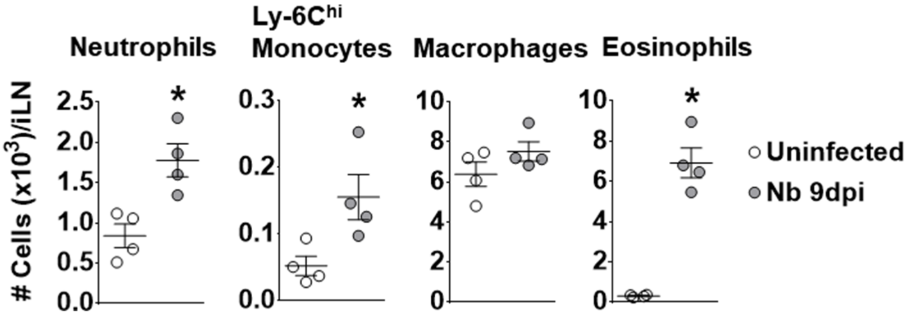

b

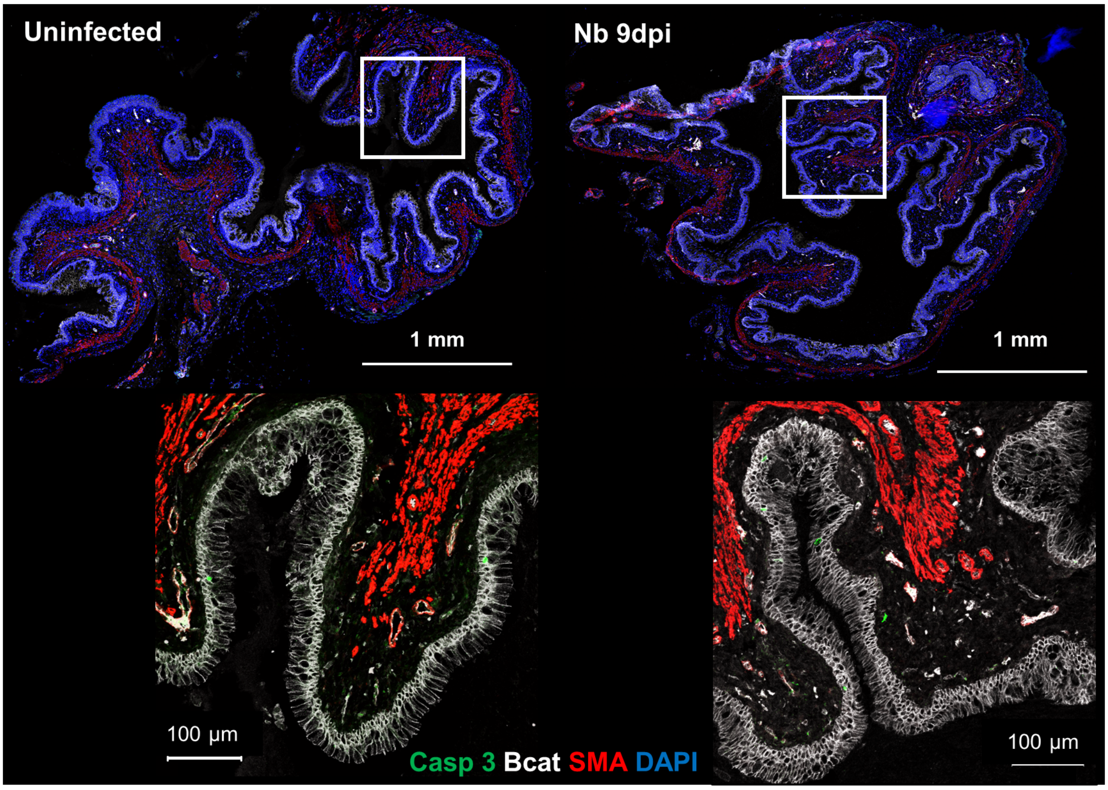

Figure S3. (a) Nb infection increases myeloid cell accumulation in the genital lymph nodes, Related to Figure 1: At 9dpi, myeloid cell populations in the iLN were analysed by flow cytometry to determine the numbers ($x10^3$) of neutrophils, Ly-6C^{hi} monocytes, macrophages and eosinophils in the iLN. Data is representative of two independent experiments with 4-5 mice per group (mean±sem). Statistical significance was calculated by Mann Whitney t test. *$p \leq 0.05$. **(b) Nb infection alone does not alter vaginal epithelial integrity, Related to Figure 1:** Female BALB/c mice were treated with 2 mg Depo Provera® (DP) 7 days prior to subcutaneous infection with Nb. At day 9 post Nb infection (Nb 9dpi), vaginal tissue (n=2) was isolated for immunofluorescent (IF) staining for beta catenin (Bcat), smooth muscle actin (SMA), 4',6-diamindino-2-phenylindole (DAPI) and cleaved caspase 3 (Casp 3). Images were taken using a 10x and 20x objective lens. White boxes indicate magnified sections. Data is representative of one experiment with 2 mice per group.

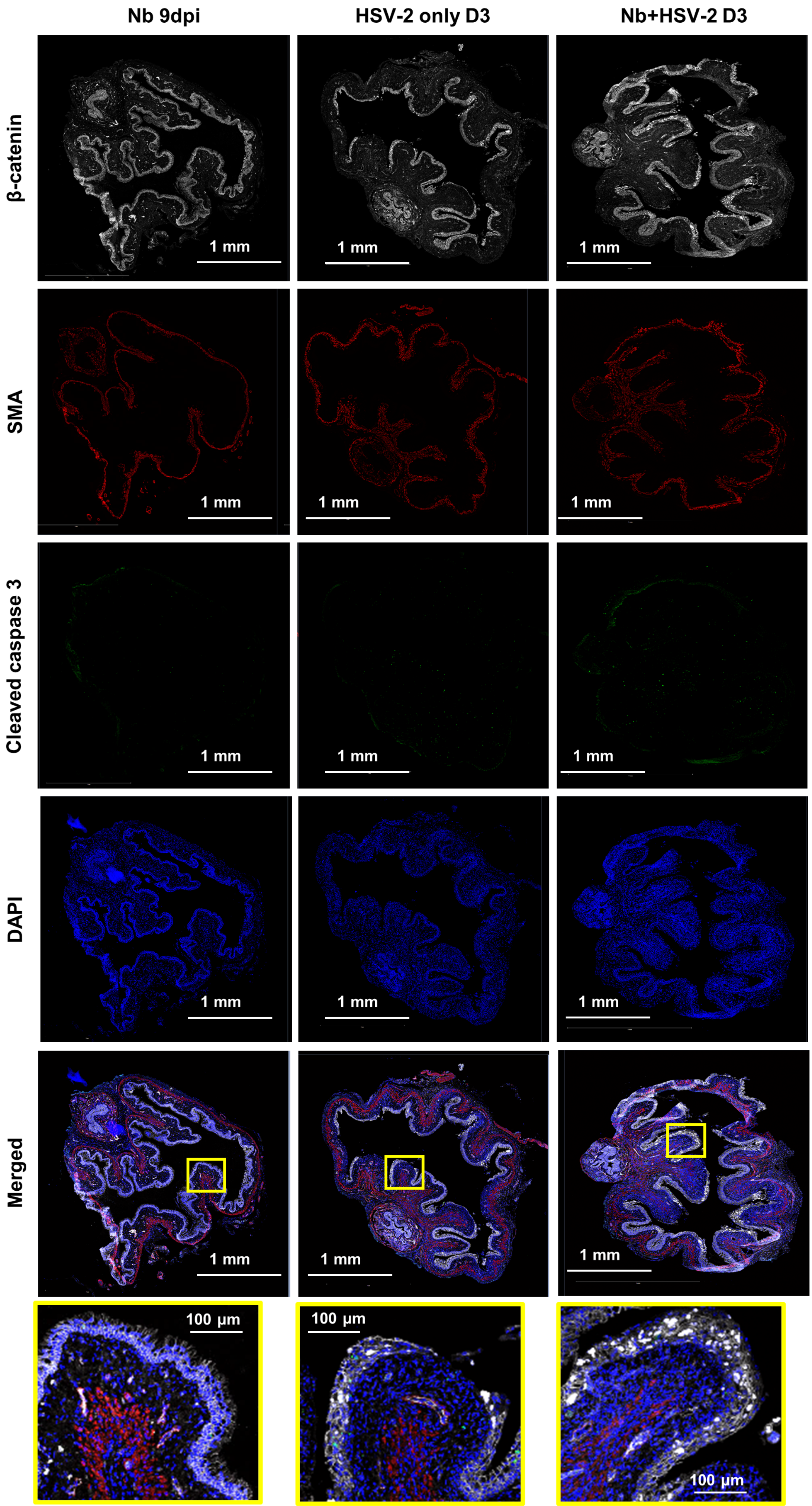

Figure S4. Virus-induced epithelial necrosis, rather than apoptosis, and early ulcer formation at day 3 post HSV-2 infection, Related to Figure 2: 7 days after Nb infection, mice were intravaginally infected with HSV-2. At day 3 post HSV-2 vaginal tissue were analysed by IF staining: beta catenin, smooth muscle actin (SMA), 4',6-diamindino-2-phenylindole (DAPI) and cleaved caspase 3. Images were taken using a 10x and 20x objective lens. Yellow boxes indicate magnified areas. Data is representative of one experiment with 4 mice per group.

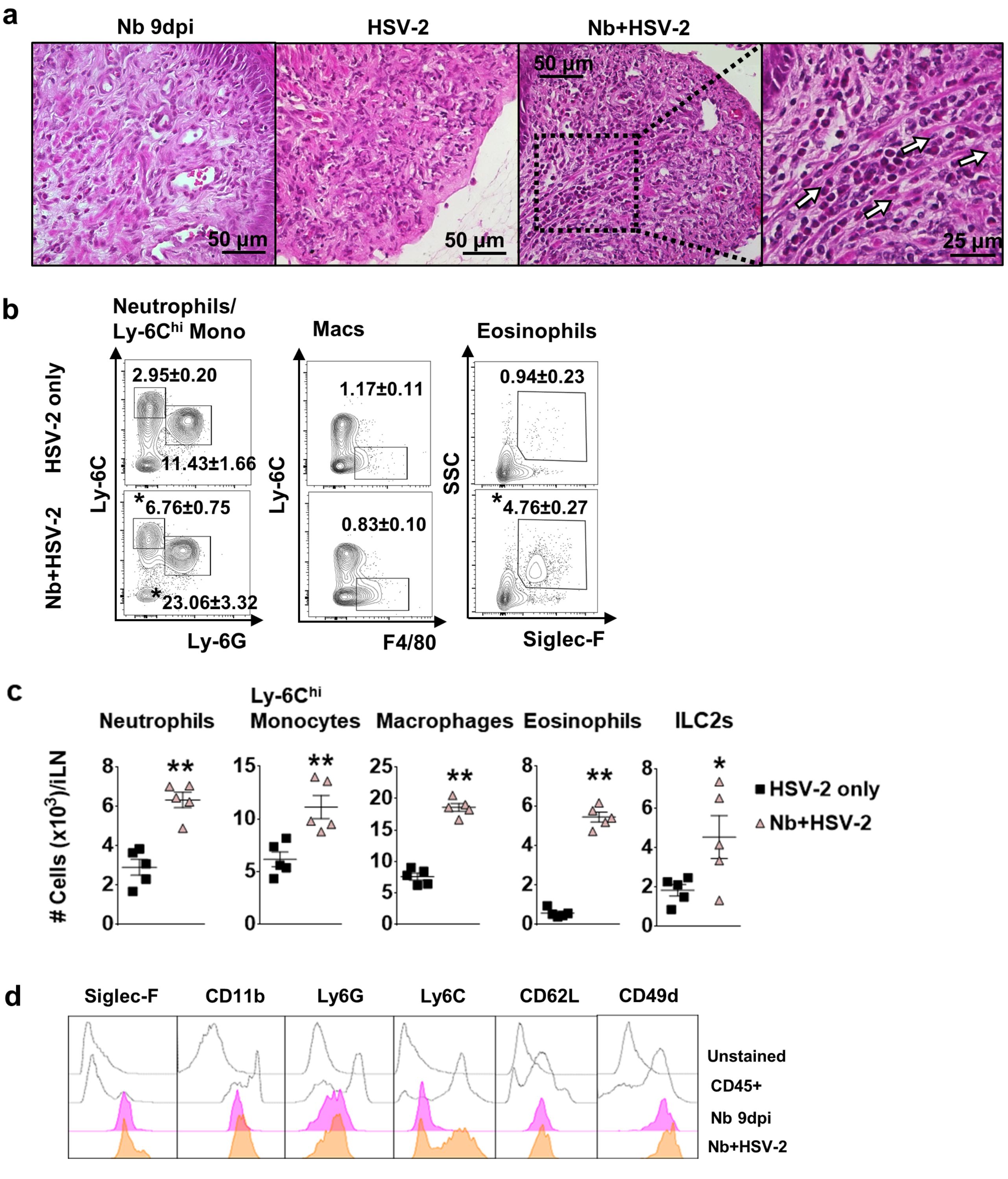

Figure S5. Prior Nb exposure increases myeloid cell inflammation in FGT and associated lymph nodes during subsequent HSV-2 infection, Related to Figure 3: At day 3 post HSV-2 vaginal tissue and iLNs were analysed by histology and flow cytometry, respectively. **(a)** Representative H&E-stained sections (n=4-5) of vaginal tissue of Nb only infected mice (day 9 PI), HSV-2 only and co-infected mice (day 3 post viral infection), displaying eosinophilic inflammation during co-infection (white arrows). **(b)** Frequencies (mean±sem) of neutrophils, Ly6C^{hi} monocytes, macrophages, and eosinophils in the FGT of HSV-2 only and Nb+HSV-2 mice at day 3 post virus infection. **(c)** Myeloid cell and ILC2 (Lin^{-} IL-7Rα^{+}ICOS^{+}ST2^{+}) populations in the iLN of HSV-2 only and Nb+HSV-2 infected mice. **(d)** Representative histograms (n=4-5) of the mean florescence intensity (MFI) of Siglec-F, CD11b, Ly6G, Ly6C, CD62L and CD49d on FGT eosinophils in Nb 9dpi and Nb+HSV-2 mice. Data is representative of two independent experiments with 4-5 mice per group (mean±sem). Statistical significance was calculated by Mann Whitney t test. *$p \leq 0.05$, **$p \leq 0.01$.

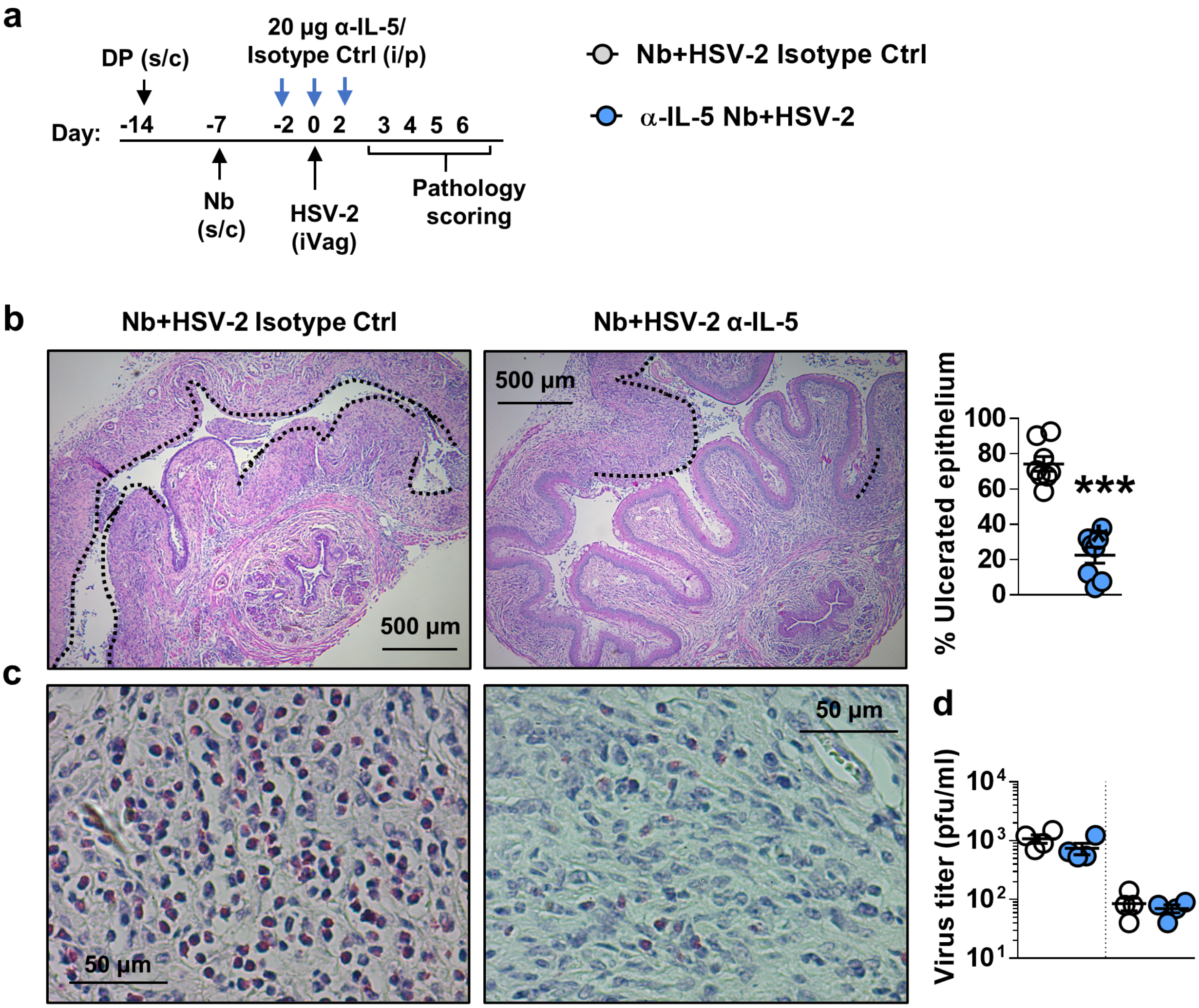

Figure S6. Anti-IL-5 antibody treatment protects against eosinophil-mediated Nb exacerbated pathology, Related to Figure 3: (a) Nb infected mice were treated with 20 µg α-IL-5 or isotype control, on day -2, 0 and 2 post HSV-2 infection. **(b)** Representative H&E-stained vaginal sections (n=4) of co-infected α-IL-5 or isotype treated mice at day 6 post HSV-2 infection. Images were taken at x50 magnification. Ulcerated vaginal epithelium is indicated by black dotted lines and qualified as percentage (%) of ulcerated epithelium. **(c)** Representative sections (n=4) of Sirius red-stained vaginal ulcers at day 6 post virus infection. Images were taken at x400 magnification. **(d)** Viral shedding was measured by plaque assay (PFU/ml) in day 3 and day 6 vaginal washes. Data is representative of two independent experiments with 4 mice per group (mean±sem). Statistical significance was calculated by Mann Whitney t test. **p* ≤ 0.05, ***p* ≤ 0.01, ****p* ≤ 0.001.

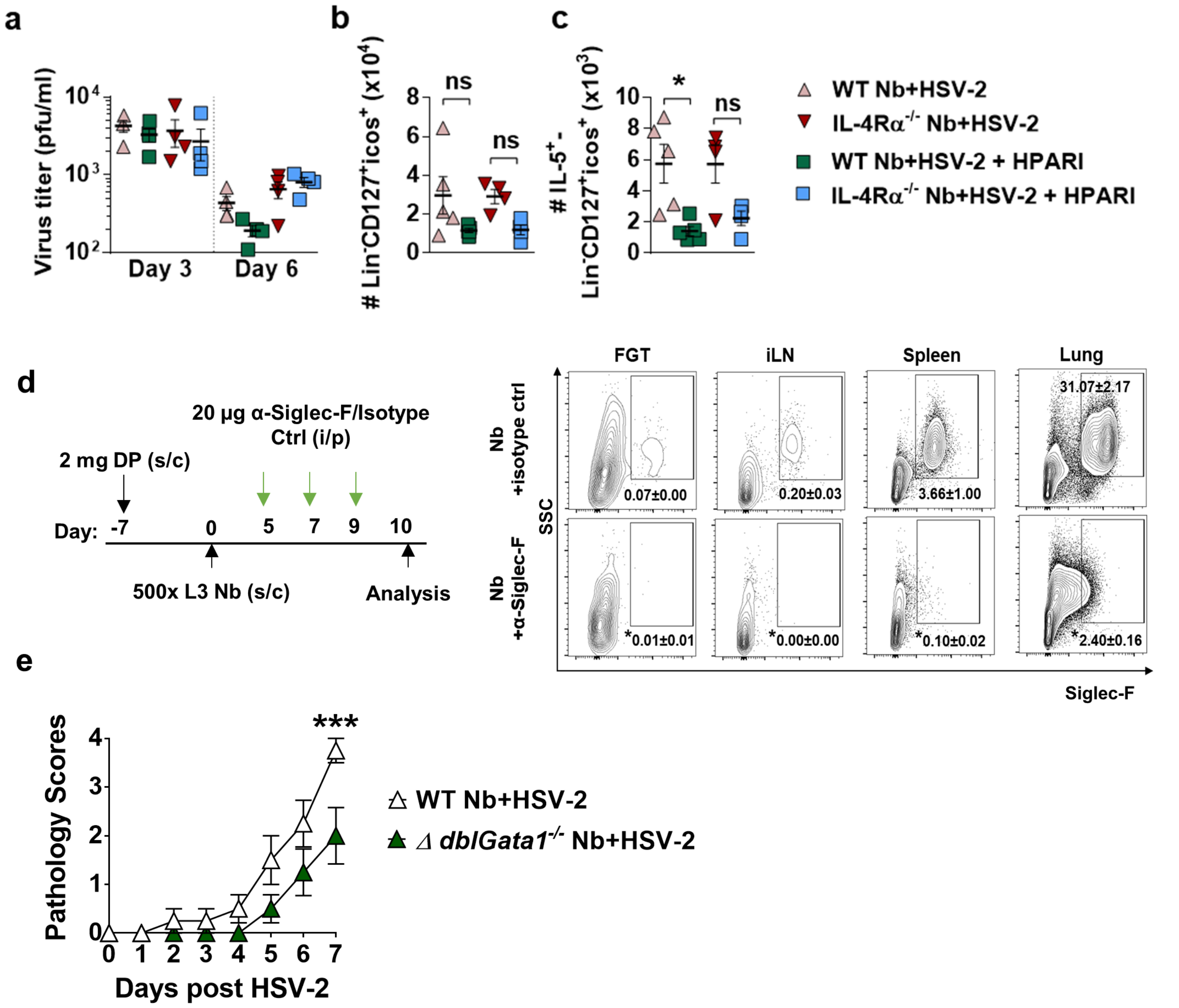

Figure S7. (a-c) IL-33 inhibition by vaginal HPARI treatment reduces IL-5 production by ILC2s, Related to Figure 5: (a) Viral shedding was measured by plaque assay (PFU/ml) in day 3 and day 6 vaginal washes. Data is representative of two independent experiments with 4 mice per group (mean±sem). Statistical significance was calculated by two-way ANOVA with Bonferroni correction for multiple comparisons. At day 6 post HSV-2 infection, FGT cells were analysed by flow cytometry: **(b)** numbers of Lin$^-$IL-7Rα$^+$ICOS$^+$ cells in the FGT and **(c)** the numbers of these cells, positive for intracellular IL-5. Data is representative of one experiment with 4-6 mice per group (mean±sem). Statistical significance was calculated by two-way ANOVA with Bonferroni correction for multiple comparisons. *$p \leq 0.05$. Related to Figure 5. **(d) Anti-Siglec-F antibody administration depletes tissue resident eosinophils, Related to Figure 6:** Nb infected mice were injected with 20 μg α-Siglec-F or isotype control, on day 5, 7 and 9 post infection. Representative flow plots (n=3) showing frequency (mean±sem) of CD11b$^+$SSC^{hi}Siglec-F$^+$ eosinophils in the FGT, iLN, spleen and lung, following antibody depletion. Data is representative of one experiment with 3 mice per group (mean±sem). Statistical significance was calculated by Mann Whitney t test. *$p \leq 0.05$. Related to Figure 6. **(e) Reduced HSV-2 pathology in co-infected *ΔdblGata1*$^{-/-}$ mice, Related to Figure 6:** Hormone synchronized WT and *ΔdblGata1*$^{-/-}$ co-infected mice were scored for virus-associated vaginal pathology following HSV-2 infection. Data is representative of one experiment with 3-4 mice per group (mean±sem). Statistical significance was calculated by two-way ANOVA with Bonferroni correction for multiple comparisons. ***$p \leq 0.001$.
